# Hypothalamic Estrogen Receptor α Is Essential for Female Marmoset Sexual Behavior Without Protecting From Obesity

**DOI:** 10.1210/jendso/bvaf012

**Published:** 2025-02-05

**Authors:** Marissa Kraynak, Molly M Willging, Daniel J Uhlrich, Robert A Shapiro, Matthew T Flowers, Karen A Manning, Sara D John, Samantha M Williams, Lukas J Henjum, Rebecca C Marrah, Hannah R Yohnk, Carter B Berg, Kevin Brunner, Ricki J Colman, Andrew L Alexander, David H Abbott, Jon E Levine

**Affiliations:** Wisconsin National Primate Research Center, University of Wisconsin–Madison, Madison, WI 53715, USA; Endocrinology-Reproductive Physiology Training Program, University of Wisconsin–Madison, Madison, WI 53715, USA; Wisconsin National Primate Research Center, University of Wisconsin–Madison, Madison, WI 53715, USA; Endocrinology-Reproductive Physiology Training Program, University of Wisconsin–Madison, Madison, WI 53715, USA; Department of Neuroscience, University of Wisconsin–Madison, Madison, WI 53705, USA; Department of Neuroscience, University of Wisconsin–Madison, Madison, WI 53705, USA; Department of Medicine, University of Wisconsin–Madison, Madison, WI 53705, USA; Department of Neuroscience, University of Wisconsin–Madison, Madison, WI 53705, USA; Department of Radiology, University of Wisconsin–Madison, Madison, WI 53705, USA; Wisconsin National Primate Research Center, University of Wisconsin–Madison, Madison, WI 53715, USA; Wisconsin National Primate Research Center, University of Wisconsin–Madison, Madison, WI 53715, USA; Wisconsin National Primate Research Center, University of Wisconsin–Madison, Madison, WI 53715, USA; Wisconsin National Primate Research Center, University of Wisconsin–Madison, Madison, WI 53715, USA; Wisconsin National Primate Research Center, University of Wisconsin–Madison, Madison, WI 53715, USA; Wisconsin National Primate Research Center, University of Wisconsin–Madison, Madison, WI 53715, USA; Wisconsin National Primate Research Center, University of Wisconsin–Madison, Madison, WI 53715, USA; Endocrinology-Reproductive Physiology Training Program, University of Wisconsin–Madison, Madison, WI 53715, USA; Department of Cell and Regenerative Biology, University of Wisconsin–Madison, Madison, WI 53705, USA; Department of Cell and Regenerative Biology, University of Wisconsin–Madison, Madison, WI 53705, USA; Department of Medical Physics, University of Wisconsin–Madison, Madison, WI 53705, USA; Wisconsin National Primate Research Center, University of Wisconsin–Madison, Madison, WI 53715, USA; Endocrinology-Reproductive Physiology Training Program, University of Wisconsin–Madison, Madison, WI 53715, USA; Department of Obstetrics and Gynecology, University of Wisconsin–Madison, Madison, WI 53715, USA; Wisconsin National Primate Research Center, University of Wisconsin–Madison, Madison, WI 53715, USA; Endocrinology-Reproductive Physiology Training Program, University of Wisconsin–Madison, Madison, WI 53715, USA; Department of Neuroscience, University of Wisconsin–Madison, Madison, WI 53705, USA

**Keywords:** ventromedial nucleus, arcuate nucleus, female sexual dysfunction, hypergonadotropism, hyperglycemia, diet-induced obesity

## Abstract

**Context:**

Estrogen receptor α (ERα) in the ventromedial (VMN) and arcuate (ARC) nuclei of female rodent mediobasal hypothalami (MBHs) provides a crucial molecular gateway facilitating estradiol (E_2_) regulation of sexual behavior, reproductive neuroendocrinology, and metabolic function. In female nonhuman primates (NHPs) and women, however, its hypothalamic counterpart remains unknown.

**Objective:**

We hypothesized that knockdown (KD) of ERα expression in the hypothalamic VMN and ARC of female marmosets would diminish sexual receptivity, while simultaneously disrupting gonadotropic and metabolic homeostasis.

**Methods:**

We ovariectomized (OVX) adult female marmosets of comparable age and weight, immediately replaced E_2_ at midcycle levels, and approximately 1 month later assigned monkeys to diet-induced obesity (DIO) within group (1) control, receiving scrambled short hairpin RNA (shRNA), or (2) ERαKD, receiving selective ERα gene silencing shRNA. Magnetic resonance imaging–guided neural surgery enabled hypothalamic infusion of viral vector shRNA and subsequent brain immunohistochemistry enabled observer-validated, NIS-elements computer software quantification of ERα knockdown.

**Results:**

ERα expression was significantly diminished in the VMN and ARC, but not the preoptic area (POA), of ERαKD females coincident with elimination of timely female sexual responses, more than 80% loss of female receptivity, modestly elevated gonadotropin levels, hyperglycemia, and diminished calorie consumption. Density and intensity of ERα-expressing cells in the VMN correlated positively with female sexual receptivity and calorie consumption, negatively with timeliness of female sexual responses, and in the ARC, correlated negatively with calorie consumption.

**Conclusion:**

ERα activation in the female NHP MBH is critically important for female sexual behavior and modestly contributes to gonadotropic and metabolic control.

Estradiol (E_2_) has been implicated as a critically important modulator of female sexual motivation in all mammalian species studied to date. In women and female nonhuman primates (NHPs), sexual motivation can fluctuate during ovulatory cycles, with highest levels occurring at mid-cycle in concert with preovulatory peaks in circulating E_2_ [1-4]. Many women with diminished serum E_2_, associated with natural or surgically induced menopause, experience diminished sexual desire [5, 6], and bilateral ovariectomy (OVX) of female rhesus macaques [7, 8] and marmoset monkeys [9, 10] is typically accompanied by reduced frequency of sexual behavior. Post-OVX estrogen replacement therapy has been shown to be effective in women and female NHPs in restoring sexual motivation to ovary-intact levels [6, 8, 9, 11, 12]. In many nonprimate species, including rodents, ungulates, and carnivores, female sexual behavior is completely eliminated following OVX and largely restored by estrogen-containing steroid replacement therapies [13, 14]. While the critical role of E_2_ in supporting sexual motivation in female mammals is well established, a fundamental understanding of molecular and cellular pathways that mediate these actions has been largely limited to mechanisms that operate in rodents. The receptors, signaling pathways, and estrogen-responsive neuronal groups that transduce E_2_ effects on sexual motivation in women, and in NHPs in particular, have remained unclear.

Female NHPs [1, 14-19], in contrast to female rodents [20, 21], do not exhibit estrus, and engage in sexual activity throughout their ovarian or menstrual cycle, while expressing more frequent displays of sexual behavior during periovulatory elevations in circulating ovarian E_2_. In rodents, on the other hand, female sexual behavior expression is closely timed with, and limited to, the fertile periovulatory period [20, 21]. Additionally, OVX-mediated E_2_ depletion in female NHPs, specifically in marmoset monkeys [9] and macaques [22, 23], has been shown to decrease, but not abolish, female sexual behavior, in contrast to rodents. Similar to female rodents, nevertheless, the hypothalamus in female NHPs is a key neural regulator of sexual behavior [24-27]. Chemical and electrolytic lesions of various nuclei within the marmoset hypothalamus have shown that, as in female rodents, the ventromedial nucleus (VMN) is an essential hypothalamic region controlling female sexually receptive behavior [24]. Complimentary studies involving electrical stimulation of, or electrophysiological recording from, the VMN in female macaques confirm the VMN as an important neuroregulatory locus of female NHP sexual behavior [26, 27]. Additional lesion and electrical manipulation studies also identify accessory roles for other hypothalamic nuclei in regulating female sexual behavior. For example, specific lesioning of the medial preoptic area (mPOA) results in elimination of proceptive, or sex-seeking/soliciting, behavior in female marmoset monkeys. Female marmoset receptivity to male sexual advances, however, still remains following mPOA lesions [25].

Studies in mice and rats have confirmed that activation of estrogen receptor α (ERα or *ESR1*) [28, 29] in the VMN is obligatory for the normal expression of sexual receptivity. Lordosis behavior, species-typical reflexive dorsiflexion of the back by which sexually receptive females enable male intromission, is absent in complete ERα gene knockout (KO) mice [30, 31], and after long-term, viral-vector–mediated ERα gene silencing in the mouse VMN [29, 32]. Estrogens may exert additional modulatory effects both through genomic and nongenomic actions mediated by ERα, estrogen receptor β (ERβ), G protein–coupled estrogen receptor (GPER), and G protein q–coupled membrane estrogen receptor (Gq-mER, aka STX receptor) in the arcuate nucleus (ARC) and mPOA [33]. The VMN, nevertheless, has been characterized as the neural locus functioning as final diencephalic output controlling downstream centers that ultimately activate spinal motoneurons to produce the lordotic posture [34-36]. By contrast, there is scant evidence available on the identity and location of ERs regulating sexual motivation in women and in female NHPs.

The receptors and neural circuitries that mediate homeostatic feedback actions of E_2_ in the primate hypothalamic-pituitary-gonadal axis likewise remain to be fully clarified. That ERα may convey negative feedback actions in the reproductive axis in primates, as it is known to do in rodents [37, 38], is supported by the finding of elevated serum gonadotropin levels in 3 women with estrogen insensitivity conferred by a loss-of-function *ESR1* gene variant [39]. The cells that express ERα and transduce these effects remain unclear. A key role for the female NHP mediobasal hypothalamus in the regulation of gonadotropin-releasing hormone (GnRH) and gonadotropin secretions was identified from the maintenance of ovulatory menstrual cycles, as well as negative and positive feedback regulation of pituitary (PIT) gonadotropin release, following surgical disconnection of the mediobasal hypothalamus in female macaques [40]. Subsequent hypothalamic lesion experiments narrowed the ovarian neuroendocrine regulation site to the ARC [41], and successive studies have implicated kisspeptin and neurokinin B neurons within the ARC as key neuropeptide regulators of hypothalamic GnRH, and thus anterior PIT gonadotropin release stimulating ovarian E_2_ release [42]. A more recent gene editing study in mice demonstrated that E_2_ uses *ESR1* in ARC kisspeptin neurons to achieve estrogen-negative feedback of the GnRH pulse generator [43]; however, it is not known if an analogous mechanism operates via ERα activation in ARC neurons of primates.

The importance of hypothalamic ERα in the regulation of body weight and metabolism in primates has likewise not been confirmed. The VMN has again been identified as a key regulatory site following electrolytic lesions of the female macaque hypothalamus [44]. When lesions included portions of the VMN, female macaques demonstrate increased weight gain. Stimulatory effects of E_2_ on energy expenditure in female rodents are transduced in ERα-expressing neurons within the VMN [45], likely by nonclassic ERα signaling [28] coupled to activation of PI3-kinase [46]. E_2_ also regulates gene expression associated with regulation of food intake and energy expenditure in the female hypothalamus, largely through ERα activation [47, 48]. Furthermore, a study by Musatov et al [45] demonstrated that viral vector–mediated ERα gene silencing in the VMN both of female mice and rats largely recapitulates a metabolic phenotype observed in whole-body ERαKO mice, including obesity, hyperphagia, impaired glucose tolerance, and reduced energy expenditure [49-51]. Deletion of *ESR1* in VMN neurons produces hypometabolism and abdominal obesity, while elimination of *ESR1* in pro-opiomelanocortin (POMC) neurons produces hyperphagia [48]. In addition, ERα-expressing neurons within the ARC have also been demonstrated to play key roles in regulating female rodent metabolic homeostasis [52]. In female primates, including women, however, the major neural receptor mechanism mediating E_2_ control of body weight and energy homeostasis signaling has not been elucidated.

The present study was thus undertaken to investigate the role for ERα-expressing neurons in the hypothalamic VMN and ARC in regulating female sexual motivation, reproductive neuroendocrinology, and metabolic homeostasis in a female NHP model. In this study, we used magnetic resonance imaging (MRI)-guided, neural infusions of an adeno-associated virus, AAV8, into the adult female marmoset mediobasal hypothalamus (MBH) to deliver a small hairpin RNA (shRNA) either encoding a scrambled virus control, with no known gene targets in this species, or an shRNA specifically designed to associate with ERα messenger RNA, alone, to prevent translation of ERα protein. We hypothesized that similar to female rodents [28, 29, 31, 45], ERα gene silencing both in the VMN and ARC of female marmoset monkeys would diminish sexual receptivity to male sexual advances, interrupt E_2_-mediated negative feedback regulation of gonadotropin release, and disrupt metabolic homeostasis. Addition of diet-induced obesity (DIO) to the study design enabled exaggeration of metabolic outcomes in the context of manipulating female sex hormone activity, as analogously employed in rhesus macaques [53], as well as in rodents and women [54].

## Materials and Methods

### Animals

Eleven adult female common marmosets (aged 2-6 years) from the Wisconsin National Primate Research Center (WNPRC) colony, and already housed in well-established male-female pairs [55], were assigned to 2 comparable groups based on age, body weight, and body mass index (Table 1): OVX + E_2_ replacement + one-to-one mixture of scrambled virus shRNA (AAV8-H1-scramb-EGFP) + virus comprising green fluorescent protein (GFP) without scrambled virus shRNA (AAV8-CMV-EGFP) (control; n = 6) and OVX + E_2_ replacement + ESR1 gene silencing shRNA (AAV8-H1-ERα34-EGFP) (ERαKD; n = 5). AAV8-CMV-EGFP was infused into controls in equal concentration to AAV8-H1-scramb-EGFP so that control monkeys received comparable titers of AAV8 viral vector compared to those infused into ERαKD monkeys since titers of AAV8-H1-scramb-EGFP, alone, were insufficient to provide parity with those of AAV8-H1-ERα34-EGFP.

**Table 1.** Selected characteristics (mean ± SEM) of adult female marmoset monkeys at study onset

Parameter	Controls	ERα knockdown
	(n = 6)	(n = 5)
Age, y	3.4 ± 0.6	3.3 ± 0.3
Body weight, g	440 ± 24	412 ± 33
Circulating estradiol, pg/mL	918 ± 146	800 ± 140
Circulating CG, ng/mL	4.2 ± 1.4	1.1 ± 0.4

Abbreviations: CG, gonadotropin; ERα, estrogen receptor α.

Female marmosets were maintained in these two groups for approximately 10 to 12 months in 0.60 m × 0.91 m × 1.83 m enclosures under 12-hour lighting, ambient temperature of approximately 27 °C and humidity of approximately 50% [53]. This study was reviewed and approved by the Vice Chancellor for Research and Graduate Education Animal Care and Use Committee of the University of Wisconsin–Madison and was performed in a manner consistent with the USDA Animal Welfare Act and regulations and the Guide for the Care and Use of Laboratory Animals. The animal care and use program at the University of Wisconsin maintains a Public Health Services Assurance and is fully accredited by AAALAC.

### Ovariectomy and Estrogen Replacement

Following baseline assessments, bilateral OVX was performed in all females at least 1 month prior to neural surgery. Cloprostenol (Estrumate, 0.75-1.50 μg intramuscular [IM] injection for 2 successive days ∼11-60 days after ovulation), an analogue of prostaglandin-F2-α, was administered prior to OVX to facilitate scheduling of OVX during the follicular phase of the ovarian cycle. Such cloprostenol administration is without subsequent effect on study outcomes [56, 57]. All females received subcutaneous (SC), E_2_-filled silastic capsules (length 29 mm, internal diameter 1.5 mm) immediately following OVX. Silastic capsules were removed and replaced at 3-month intervals post OVX. Approximately 1 month prior to neural surgery, the number of implanted, E_2_-filled capsules was increased to 4 per monkey to approximate mid-cycle, periovulatory systemic E_2_ levels and to maintain negative feedback regulation of circulating PIT gonadotropin levels within the range of ovary intact female marmosets [56, 57] (see Table 1).

### Efficacy of RNA Interference Activity for Short Hairpin RNA Specific to ESR1

An shRNA effective at KD ERα protein expression was identified employing an in vitro approach using MCF7 cells with subsequent in vivo confirmation of efficacy using 4 adult female marmoset monkeys.

#### In vitro

Two shRNAs were initially designed to KD *ESR1*, 1) ERα34 shRNA and 2) ERα56 shRNA, and thus KD ERα protein expression. The Dharmacon shRNA design center online (https://horizondiscovery.com/en/dharmacon) was employed to design the ERα34 shRNA based on the small interfering RNA (siRNA) sequence CCTACTACCTGGAGAACGA. Candidate shRNAs were advanced to the next round only if the chosen sequence was identical in marmoset monkeys, human, and mouse. Furthermore, candidate shRNAs were required to target a region of *ESR1* that was common to all isoforms. The ERα56 shRNA used the siRNA sequence GGCATGGAGCATCTGTACA, which was based on the sequence used by Musatov and colleagues [32] to KD mouse ERα protein expression, except one nucleotide was changed to match the marmoset and human *ESR1* sequence. Additionally, a scrambled control shRNA was generated based on the siRNA sequence GAACGACGACATGTCTACC, which maintained the same nucleotide composition as the ERα34 shRNA, but lacked ERα KD capability. Candidates were further scrutinized by examining potential off-target effects using BLAST analysis against the database for marmoset monkeys [58]. All shRNAs used the same loop sequence CTTCCTGTCA based on that used by Musatov and colleagues [32].

**Figure 1.**
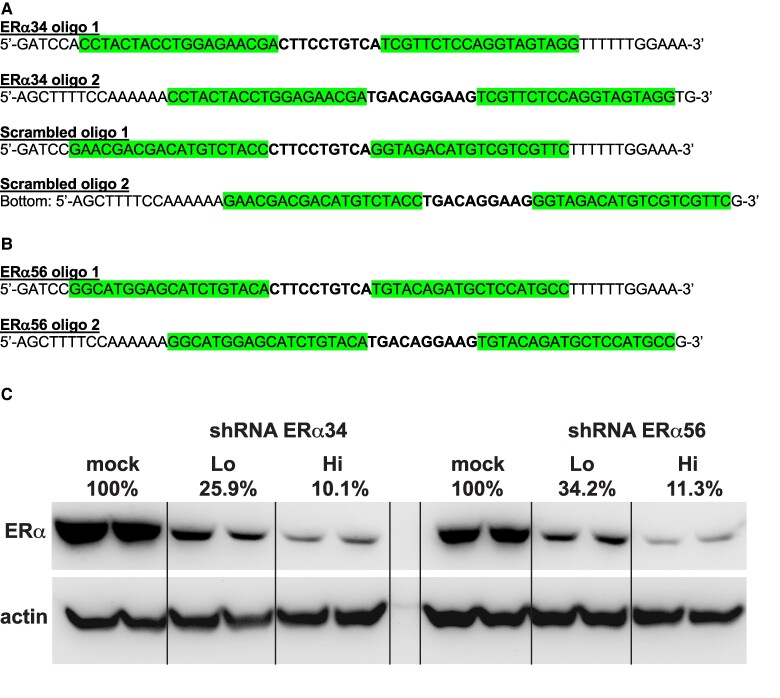
Adeno-associated virus 8 (AAV8)-mediated short hairpin RNA (shRNA) reduces ERα protein expression in vitro. Schematic representations of the shRNA sequences packaged within AAV8 vector infused into ERαKD adult female marmoset monkey hypothalami in this study, A, ERα34 shRNA and scramble control shRNA, and B, the alternative ERα56 shRNA packaged within AAV8 and not employed in this study. Highlighted text illustrates corresponding small interfering RNA (siRNA) sequences and bold text indicates loop sequences. C, Western blot analysis illustrating diminished ERα protein expression in MCF7 transfected with the shRNA-expressing vectors pAAV-H1-ERα34-EGFP or pAAV-H1-ERα56-EGFP compared to mock transfected cells treated with Lipofectamine alone. The consistent expression of actin, the protein loading control, across all lanes indicates diminished ERα expression is due to shRNA knockdown. %, concentration of Lipofectamine; Lo, Hi, relative concentrations of shRNA plasmid; Mock, transfected cells treated with Lipofectamine alone.

We first obtained the predesigned pAAV-H1-EGFP (green fluorescent reporter gene) plasmid from Vector Biolabs and then cloned in the shRNA sequences that we designed into the 5′ BamHI (GGATCC) and 3′ HindIII (AAGCTT) restriction sites (Fig. 1A and 1B) to generate 3 viral vectors: pAAV-H1-ERα34-EGFP, pAAV-H1-ERα56-EGFP, and pAAV-H1-scramb-EGFP. All shRNA plasmids used the H1 promoter to drive shRNA expression and expressed GFP from a separate cytomegalovirus (CMV) promoter, thus enabling identification of GFP-expressing hypothalamic cells infected during subsequent neuroimmunohistochemical morphological analyses. We transfected the shRNA-expressing plasmids into MCF7 cells (a human breast cell line with estrogen, progesterone, and glucocorticoid receptors [59]) using Lipofectamine (Invitrogen) transfection reagent at 2 different doses compared to mock transfected cells treated with Lipofectamine alone. ERα protein expression was quantified by Western blot using the 6F11 mouse monoclonal antibody (Thermo Fisher Scientific catalog No. MA1-27107, RRID:AB_780508, https://www.antibodyregistry.org/AB_780508) [60] to assess the relative ability of each shRNA to interfere with *ESR1* expression. pAAV-H1-ERα34-EGFP and pAAV-H1-ERα56-EGFP both elicited strong KD of ERα protein (Fig. 1C). Vector Biolabs performed all recombinant AAV production, using capsid serotype AAV8 and inverted terminal repeat serotype AAV2, to generate AAV8-H1-ERα34-EGFP, AAV8-H1-ERα56-EGFP, and AAV8-H1-scramb-EGFP. AAV8-H1-ERα34-EGFP was chosen for in vivo ERα KD experiments due to its higher titer relative to AAV8-H1-ERα56-EGFP.

#### In vivo

Prior to the onset of the 10- to 12-month study, and using surgical and neuroinfusion procedures described later, 3 adult female marmosets were infused with AAV8-H1-ERα34-EGFP expressing shRNA targeting *ESR1* for in vivo RNA interference (RNAi) (unilateral infusion, n = 2; bilateral infusion, n = 1) and 2 were infused with AAV8-H1-scramb-EGFP expressing a control scrambled shRNA (unilateral infusion, n = 1; bilateral infusion, n = 1). After 8 to 23 and 12 to 13 days, respectively, marmoset monkeys were euthanized, hypothalami harvested at necropsy, and subsequently processed for immunohistochemical visualization of ERα protein expression. As anticipated, in the 3 marmosets receiving unilateral or bilateral infusions of shRNA targeting *ESR1*, scant expression of ERα protein was observed. In contrast, ERα protein was robustly expressed in the 2 marmosets infused with scrambled shRNA.

### Neural Infusion Surgery and Estrogen Receptor α Knockdown

OVX adult female marmosets received bilateral stereotaxic infusions into the hypothalamic VMN and ARC of AAV8-H1-ERα34-EGFP expressing shRNA targeting *ESR1* for in vivo RNAi, and thus diminishing *ESR1* translation into ERα protein (ERαKD, n = 5), or an AAV8-H1-scramb-EGFP expressing a control scrambled shRNA (control, n = 6). To match the titer of the AAV8-H1-ERα34-EGFP virus, control animals received an infusion mixture of AAV8-H1-scramb-EGFP and AAV8-CMV-EGFP (Vector Biolabs) at a final viral particle ratio of 1:1. The latter did not express shRNA. Study onset (0 months) was the day of these hypothalamic viral vector infusions and neurosurgery.

**Figure 2.**
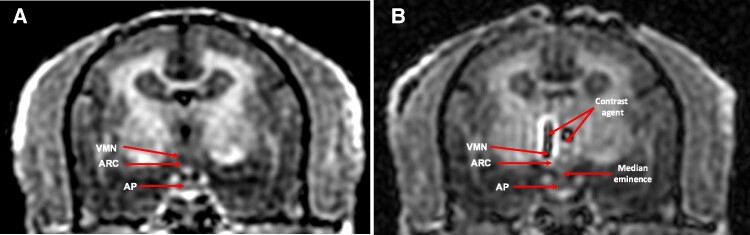
Representative magnetic resonance imaging (MRI) T1 coronal sections of the brain and head of an ERαKD adult female marmoset, ERαKD 2, A, before and B, during MRI-guided bilateral infusion of viral vector comprising short hairpin RNA (shRNA) specific for ESR1 and a gene promoter for GFP (AAV8-H1-ERα34-EGFP) into the mediobasal hypothalamus. Infusions contained MRI contrast agent and thus manifest as light gray-black–appearing mixtures (arrows, contrast agent). Each coronal image is taken at a similar rostral-caudal location at the level of the median eminence-pituitary stalk, and arrows indicate the anatomical locations of the ventromedial nucleus (VMN) and arcuate nucleus (ARC). The tissue-dense posterior pituitary is visualized as light gray immediately below the median eminence and darker anterior pituitary (AP).

Since accurate, within-brain placement of viral vector was essential for gene silencing efficacy and specificity, we used a single, presurgical, neuroanatomical MRI scan to identify the unique locations of each VMN and ARC within each marmoset, refined and adapted from previous studies [61]. To accomplish this, each female marmoset was food-deprived overnight, sedated with an IM injection of ketamine (10 mg), analgesia was provided with an IM injection of meloxicam (2 mg), 2 mL isotonic saline was injected SC to provide fluid administration and atropine (0.016 mg) was administered IM to reduce salivation and bronchial secretions before surgery, followed by placement of the marmoset into an MRI-compatible stereotaxic frame. Lateral placements of both left and right earbars, as well as vertical placements of palate and left and right eye bars, were all confirmed. Three-dimensional coordinate locations of both left and right VMN and ARC were subsequently obtained from a T1 MRI anatomical scan adapted for marmosets [62] in a next-generation 3T MRI system MR750 (GE Healthcare) and performed at least 3 days prior to viral vector infusion (Fig. 2).

Each presurgical scan employed a 3-inch (∼7.6 cm) surface coil and comprised 3-dimensional (3D) T1-weighted images with an inversion-recovery (IR) prepped, fast gradient-echo (IR-fGRE) sequence adapted for marmosets [62]. Scanning parameters included an inversion time of 450 ms, repetition time of 6.8 ms, echo time of 2.9 ms, and receiver bandwidth of 62.5 kHz. The matrix size was 256 × 256 × 128 (X × Y × Z). The 3D T1-weighted scans produced 248 coronal 0.3-mm-thick contiguous slices. Using coronal slices incorporating the hypothalamus from each monkey's presurgical scan, we used contrast-agent filled earbars in the stereotaxic frame to provide a rostral-caudal (RC) zero plane (Z coordinates), the middle of the third ventricle at the level of the MBH to generate a medial zero plane (X coordinates), and the farthest lateral point of each earbar to provide a dorso-ventral zero plane for right and left sides separately (Y coordinates). Based on these 3D parameter sets, target coordinates for entry into the skull, as well as for rostral, medial-rostral, medial-caudal, and caudal left and right VMN viral vector infusion sites, and analogous infusion locations for both left and right ARC, were estimated based on marmoset neuroanatomy atlas coordinates [61].

For viral vector infusion surgery, each marmoset was sedated and prepped as described for their presurgical MRI scan. In addition, each monkey was intubated, and the anesthesia was continued under isoflurane (1%-2%). Each marmoset was repositioned into similar stereotaxic frame parameters as noted during their presurgical MRI scan. Vital signs (heart rate, blood oxygen, respiration, and temperature) were regularly monitored during the procedure, and the animals were wrapped for warmth. A series of small-volume, SC injections of lidocaine (total, 1 mg) were given along the anticipated incision site to provide local anesthesia. An approximately 30-mm incision was made on the top of the animal's head. Stereotaxic coordinates, estimated from our presurgery anatomical MRI scan, guided calculation of the insertion sites of a 22-gauge guide cannula housing a 28-gauge stylet to target the VMN and ARC in the hypothalamus. The estimated midline location above the medial infusion sites for both VMN and ARC was marked on the skull to enable excision of an approximately 10-mm diameter portion of skull using a surgical bone drill. A micromanipulator, with cannula holder and guide cannula with screw-in solid stylet, were placed on the stereotaxic frame to confirm midline of the brain from the medial location of the superior sagittal sinus, and presurgical scan estimated target coordinates for left VMN and ARC sites were revised accordingly. A 27-g needle, customized into a miniature scalpel blade, was used to open an approximately 5-mm incision in the dura mater vertically above the anticipated locations of the VMN and ARC infusion sites on the left side of the marmoset brain, and avoiding the left lateral extent of the superior sagittal sinus.

At a trajectory angle of 0° (vertical), a 22-gauge guide cannula housing a sterile 28-gauge stylet was lowered into the medial-rostral estimated extent of the left ARC, as modified for marmosets [63, 64]. The stylet was then replaced by a 28-gauge infusion cannula attached to a Hamilton syringe controlled by a programmable infusion pump. A solution of viral vector (∼2.4 × 10^10^ packaged genomic particles of AAV8 in 3.0 μL of sterile saline [Amresco, Avantor International LLC]) mixed with gadolinium-containing Multihance MRI contrast agent (2-mM gadobenate dimeglumine, Bracco Diagnostics Inc), was infused at 0.2 μL per minute. All subsequent infusion sites on both sides of the brain received this identical 3.0 μL infusion. The AAV8 virus used in this study was shown to readily infect neurons [65, 66] and was without notable inflammation in the NHP brain [64]. To minimize dorsal tracking of viral particles and contrast agent when the infusion cannula was raised, there was a 5-minute delay following cessation of infusion before removal of the infusion cannula alone to permit its flushing of sufficient fluid while outside the brain to confirm infusion integrity of the system. The stylet was repositioned inside the guide cannula. Both guide cannula and stylet were then gradually removed from the brain. Superficial skin sutures were placed using 4/0 Vicryl (polyglactin 910) suture to temporarily close the incision site during transfer to the MRI scanning room while the marmoset remained in the stereotaxic frame.

The contrast agent remaining at the medial-rostral ARC infusion site was used to confirm accuracy of the infusion site location from a mid-surgery, approximately 23-minute MRI T1 scan obtained approximately 10 to 20 minutes following the first infusion on the left side. When necessary, presurgical scan estimated target coordinates for left VMN and ARC sites were revised. Guide cannula, stylet, and infusion cannula insertion, infusion, and removal procedures were then repeatedly performed to enable AAV8 infusion into rostral, medial-rostral, medial-caudal, and caudal VMN and ARC infusion sites on the left side of the brain.

Following all left-side infusions, a 27-g needle customized into a miniature scalpel blade was used to open an approximately 5-mm incision in the dura mater vertically above the anticipated locations of the VMN and ARC infusion sites on the right side of the marmoset brain, avoiding the right lateral extent of the superior sagittal sinus. A similar series of procedures, infusions, and MRI scan were performed on the right side of the brain analogous to those performed on the left. When necessary, adjustments to presurgical estimates for rostral, medial-rostral, medial-caudal, and caudal right VMN and ARC infusion sites were made following the second within-surgery MRI scan.

### Immunohistochemical Processing of Marmoset Brain for Visualizing and Quantifying Green Fluorescent Protein and Estrogen Receptor α Protein Expression

Following upper body perfusion with 4% paraformaldehyde (pH 7.6), the brain was removed, post-fixed at 4 °C for an additional 12 to 16 hours, and cryoprotected in graded (10%-30%) sucrose/phosphate-buffered saline (pH 7.2) solutions. Each postfixed brain was placed in a customized plastic mold designed to accommodate the dorsal surface of an adult marmoset brain, thus exposing ventral surfaces landmarks and enabling placement of mold-guided, microtome blades cutting the brain into coronal plane portions. The whole brain was blocked coronally, removing the portions rostral to the optic chiasm and caudal to the mammillary bodies. The remaining block was cryosectioned at 40 μm in the coronal plane to produce a series of sections spanning the entire RC extent of the hypothalamus from the rostral mPOA to the most caudal part of the ARC.

Neuroimmunohistochemistry procedures for GFP and ERα were performed and adapted for the marmoset monkey [60, 67]. Sections at regular intervals were immunostained for ERα using the mouse monoclonal antibody 6F11. DAB-staining was subsequently used to reveal specific primary antibody binding. Sections near those selected for ERα immunostaining were stained for GFP using the rabbit polyclonal antibody AB3080 (Millipore catalog No. AB3080, RRID:AB_91337, https://www.antibodyregistry.org/AB_91337) [67]. Additional series of sections were processed without the primary antibody to assess nonspecific staining.

### Confirmation of Neural Targeting Accuracy From Neuroimmunohistochemical Analyses of Green Fluorescent Protein Expression in the Mediobasal Hypothalamus

Digital images from coronal brain sections immunostained for GFP were obtained with a Nikon Microphot-FA microscope using a QImaging Retiga 200R CCD camera and Nikon NIS Elements software. Most images for analysis were obtained using a 4× objective. Each tissue section picture was the composite of several images stitched together by the Nikon system during the photo acquisition process. This resulted in a series of coronal images through the hypothalamus from the rostral POA at the level of the optic chiasm to the mammillary bodies in the caudal hypothalamus, thus caudal to the ARC.

### Quantitative Analysis of Estrogen Receptor α Gene Knockdown Achieved in the Mediobasal Hypothalamus

ERα-immunoreactive (ir) labeled nuclei within cells of the POA, VMN, and ARC regions of interest (ROIs) within the mediobasal hypothalamus, together with discrete subregions VMN-mid (VMNm), VMN-ventral (VMNv), ARC-dorsal (ARCd), and ARC-ventral (ARCv), were quantified in digital images of coronal hypothalamic sections employing measures of cell counts (density, D) and normalized labeling intensity (I). Labeled cells were detected using the NIS Elements Advance Research Dark Spot Detection feature. The contrast threshold in the detection algorithm was determined for each case by adjusting cell counts derived from the spot detection algorithm to match cell counts made by 3 observers on identical images of approximately 6 sections per ROI. Observer counts differed by less than 15% (mean, 8.2%), NIS algorithm threshold parameters were adjusted so that NIS-derived cell counts fell within 5% of the average counts of the 3 observers. The NIS Elements AR software calculated the mean pixel intensity of each identified cell, and we used this to derive the intensity of ERα staining. The average pixel values of the observer-derived threshold cells determined the low (lighter) end of the intensity staining range. The high (darker end) of the range was determined by the average pixel intensities of the darkest 2% of labeled cells, typically in the POA or PIT, which were not targeted by the infusion. The staining intensity of individual cells was normalized (from 0 to 1) within the light to dark intensity range.

The number of labeled ERα cells was quantified in terms of cell density per unit area. This was determined by counting the number of immunostained neurons within ROIs of 1000 μm × 500 μm ellipses (areas of 0.395 mm^2^) positioned within the POA, VMN, and ARC, in addition to ROIs within the VMNm and VMNv as well as ARCd and ARCv for all 9 female marmosets. For each hypothalamic ROI within each female, we used the average of 3 sections with the highest density of labeled cells as the density measure for that ROI. One exception, however, involved the rostral POA, the area with typically the most ERα expression, that was lost during processing in one control female, M1. The POA ROI density measures from control female M1 were thus obtained from a typically more modest, ERα-immunostained portion of the POA. Since this POA ROI density value for ERα was not an outlier in the context of the remaining 8 females, this monkey's ERα data were retained for quantitative analyses.

### Technical Note Regarding Effects of Focus Plane on Cell Quantification

Cells appear most darkly labeled when the microscope objective is focused at the tissue depth that contains the cell. When the focal plane deviates from the depth of the cell, the cell blurs, and its staining darkness declines. To minimize this confound, images were collected when the microscope objective was focused on the middle depth of the tissue. While this will lead to underestimating the darkness of label of a cell located above or below the middle of the tissue section and possibly prevent its detection, we found this effect to be small when using a 4× objective with its relatively large depth of field.

To determine the effect of focus on estimates of staining intensity and cell count, we systematically varied focus across the depth of a 40-μm-thick tissue section from a control animal and collected images through a large group of labeled cells. We applied the NIS analysis to the images and found that an image captured with a 4× objective focused in the center depth of the tissue provides a reliable estimate of the number of cells and their staining intensity. On average, mid-depth focus captured more than 95% of cells that were identified at all the focal positions. The additional cells were stained lightly, near detection threshold. To examine focus effects on estimates of staining intensity, we tracked the mean pixel intensity of all identified cells across the depths of focus. The average difference between the estimate of staining darkness at mid-depth focus and the maximum darkness at any depth, normalized to the full range of staining intensities, was approximately 0.021 ± 0.027 std. The average normalized intensity of labeled neurons in the VMN and ARC of control animals that received the scrambled shRNA was 0.326 (in a 0 to 1 assessment system), so the effect of off-focus imaging was relatively small.

### Hormone Determinations

Several hormone concentrations were determined in monthly collections of plasma samples. For steroid hormone analyses, including dehydroepiandrosterone (DHEA), androstenedione (A_4_), testosterone, estrone (E_1_), and E_2_, plasma aliquots underwent extraction [68] and were subsequently submitted for analysis on a QTRAP 5500 quadruple linear ion trap mass spectrometer (AB Sciex) equipped with an atmospheric pressure chemical ionization source (liquid chromatography–tandem mass spectrometry [LC-MS/MS]) [56]. Plasma chorionic gonadotropin (CG) levels (New World primate equivalent of luteinizing hormone, LH) [69] were determined by a validated radioimmunoassay [70], detection limit 0.67 ng/mL (antibody catalog No. 518B7, RRID:AB_2756886, https://antibodyregistry.org/search.php?q=AB_2756886). Intra-assay and interassay coefficients of variation were 15.6% and 7.9%, respectively.

### Behavioral Observations

Following study onset, the 9 male-female pairs were acclimated to the testing cages, as previously described [55]. At least 5 months after study onset, male and female pair-mates were placed in single housing for 30 days prior to behavioral testing. During the separation period, pairs remained out of visual and tactile contact with each other. Following 30 days of single housing, and while remaining singly housed, male-female pairs were placed in a behavior testing cage for 3, 30-minute testing sessions per week for 2 weeks. During testing, males were placed in a holding box for 5 minutes before being allowed into the main testing cage with the female. Quantitative observations were digitally recorded and manually scored on to a laptop by at least 2 observers following a previously validated ethogram [55]. Interobserver and intraobserver reliability were 80% or greater.

To identify and quantify female marmoset sexual receptivity in detail, we used only digital recordings. This enabled identification and quantification of an additional, previously unrecognized behavioral indication of female marmoset receptivity. Prior to this, no specific receptive posture response to a male mount had been reported for female marmosets, in contrast to many female NHPs, such as macaques, when females exhibit a quadrupedal stance supporting the full body weight of the mounted male [71]. When receptive to a male marmoset mount, and while clinging on to vertical substrate (a common location for this naturally arboreal NHP), female marmosets move their feet in a posterior direction, usually downward, away from their hands, widening their vertical stance, and more importantly, repositioning their anogenital region to be more dorsal facing, enabling penile intromission. Female vertical stance was defined as extending from the top of the fingers to the top of the toes, while holding on to the vertical cage mesh, and averaged for both left and right sides. Female vertical stance was quantified immediately prior to a male mount and compared to that during a male mount with or without intromission. Vertical stance measures were obtained using digital calipers on frozen screen images, and only when both hands and feet were observed. Male marmoset mounts of either control or ERαKD females resulted in penile intromission only when females widened their vertical stance, as just described, by at least 0.5 cm (control: 1.6 ± 0.5 cm [mean ± SEM], ERαKD: 1.8 ± 0.9 cm). Ejaculation commonly occurs during a single intromission in marmosets [72]. Female marmoset receptive vertical stance postures do not appear reflexive, and thus is unlikely to be an NHP equivalent of the well-classified reflexive receptive posture of lordosis exhibited by estrous female rodents to readily enable a mounted male to intromit and ejaculate.

Behavioral sequence analysis was used to identify statistically significant sequences of sexual behaviors observed between each of the 9 male–female pairmates and the consequences of E_2_ treatment manipulation to disrupt marmoset-typical behavioral transitions during sexual interactions, as previously described [57]. A behavioral transition included any behavior that followed within 10 seconds of a previous behavior. For example, when “male mount female” is the initiating behavior, well-established male–female marmoset pairmates commonly transition from there to either “female reject mount” or “female receptive posture.” Whichever occurred would be scored as a single, “2-component” behavioral transition.

Contingency tables and chi-square test statistics were used to analyze the probability of each “2-component” transition occurring within a female treatment group. As previously described [55], frequencies of initial behaviors and transitions were tabulated from all testing sessions and used to determine the expected frequency occurrence of each behavioral transition. Five behavioral transition sequences were thus identified. Chi-square statistics were generated for each transition for each female treatment group in the context of the number of observational hours and compared with the expected frequency of the transition generated [57]. The higher the chi-square value, the more likely the transition was a statistically significant (*P* < .05) behavioral sequence.

### Diet-induced Obesity From Consumption-driven Weekly Increments in Daily Calories

Monkeys were fed a Mazuri Callitrichid High Fiber Diet #5MI6 (Purina Mills International), providing 53% carbohydrate, 20% protein, 6% fat, and 10% fiber by weight, with a metabolizable energy of 3.3 kcal/g (∼61%, 23%, and 16% kcal from carbohydrate, protein, and fat, respectively) [73]. Following study onset, total daily calorie consumption by each male-female study pair was recorded. To achieve DIO, diet allotment for each male-female study pair was increased weekly by approximately 66 kcal per day (equivalent to 20 g diet wet weight/day) if the entire daily allotment had been consumed during at least 4 out the 7 previous days, as previously validated [57].

### Assessment of Daily Calorie Consumption While Maintaining Diet-induced Obesity

At 7 months post OVX, each female was singly housed in a marmoset housing room, but outside visual contact with her male pairmate, while still maintaining vocal and auditory contact. Each female's daily calorie allotment began at 50% of those provided when housed with their male pairmate. Dietary allotment was increased weekly by approximately 33 kcal per day (∼10 g diet wet weight) only when the entire daily allotment was consumed during at least 4 out the 7 previous days. The total kcal consumed daily were recorded for each female for 8 weeks during 8 to 9 months post OVX.

### Body Composition

Animals were weighed weekly. Area under the curve (AUC) assessment of weight over time, calculated by the trapezoidal rule, was employed to better detect recurring differences in weight gain, as previously employed [74]. At baseline, as well as at 3 and 6 months post OVX, total body composition was assessed by dual-energy x-ray absorptiometry (DXA, iDXA, GE/Lunar Corp) on animals sedated with ketamine (up to 40 mg/kg, IM) followed by dexmedetomidine (up to 50 µg/kg, IM). Fat, fat-free mass (FFM) (excluding bone), bone mineral content (BMC), and bone mineral density (BMD) were determined for total body as well as previously validated ROIs, including abdomen, chest, thighs, lower legs, and arms [57].

### Locomotor Activity

At 6 months post OVX, a small accelerometer (Actiwatch Mini, CamNtech Ltd) was added to each female's standard collar. Activity and intensity of movement were recorded every 30 seconds over an approximately 2-week period after which the accelerometers were removed. For the most part, activity recorded represented whole-body movements, and not limb or head movements, alone [75]. Accelerometer data were averaged for every hour, day (during lights on), night (during lights off), morning (0600-1200 hours), afternoon (1200-1800 hours), and 24 hours. AUC activity values were also assessed to detect recurring differences in activity over time.

### Fasting Glucose and Oral Glucose Tolerance Test

Fasting glucose and glucoregulation were assessed in overnight-fasted, awake animals. Fasting glucose was determined at baseline and 6 months post OVX, while glucoregulation was assessed by OGTT only at 6 months following OVX. Following a baseline blood sample at the onset of the OGTT, marmosets were given an oral dose (5 mL/kg) of 40% sucrose. Blood samples were then collected at 15, 30, 60, and 120 minutes (2 hours) following sucrose administration and assessed for glucose [57]. Glucose was measured by glucometer (Accu-Check Aviva, Roche Diagnostics). AUC glucose values during the OGTT were also assessed to detect between-group differences in accumulating high levels of glucose over time.

### Statistical Analysis

Data collected were analyzed using SPSS software. ERα cell nucleus immunostaining intensity, and density of ERα-labeled cells within ROIs, as well as behavioral, endocrine, and somatometric parameters, were analyzed using independent *t* tests to compare means. Plots are expressed as means ± SEM. Steroid and CG hormone data were log-transformed, and % behavioral data were transformed to arcsin, prior to analysis of variance or correlation tests. Linear regression was performed to detect relationships within mediobasal hypothalamic ROI immunostaining quantified for ERα cell density vs ERα cell intensity. Multiple linear regression analyses were performed to detect relationships between mediobasal hypothalamic ROIs for quantified ERα cell density immunostaining separately from those of ERα cell intensity immunostaining, as well as between quantified ERα cell density immunostaining in mediobasal hypothalamic ROIs and quantified behavioral, endocrine, and metabolic parameters.

## Results

### Targeted Gene Knockdown of Estrogen Receptor α Within Ventromedial Nucleus and Arcuate Nucleus Regions of Interest of the Mediobasal Hypothalamus in Estrogen Receptor α Knockdown Female Marmosets

**Figure 3.**
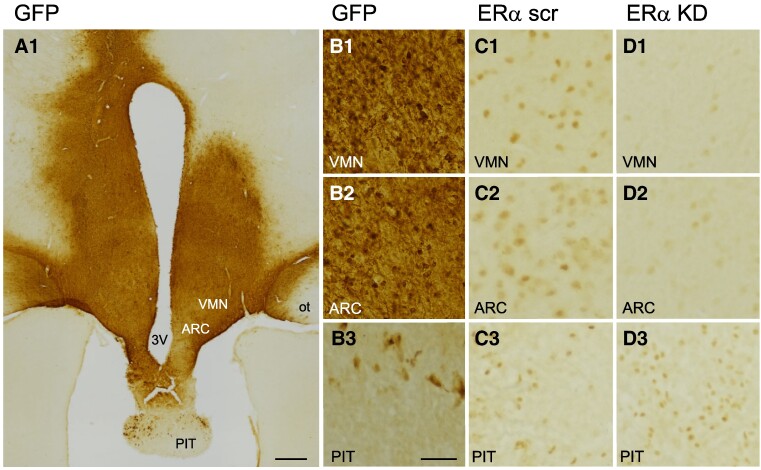
Green fluorescent protein (GFP) and ERα immunohistochemical labeling following hypothalamic infusion of adeno-associated virus 8 (AAV8) construct. A1, Low-power image of immuno-label for GFP in perihypothalamic and hypothalamic areas that demonstrates extensive bilateral viral vector infection of cells in the vicinity of the third ventricle (3V) hypothalamic ventromedial nucleus (VMN) and arcuate nucleus (ARC), with a modicum of infection in the median eminence (ME), dorsal anterior pituitary (PIT), and medial portions of both optic tracts (ot). B1 to B3, Higher-power images show GFP immunostaining in cell bodies and processes in VMN, ARC and PIT, respectively. C1 to C3, Higher-power images illustrating ERα-labeled nuclei from a scrambled control female marmoset (ERα scr) that demonstrate abundant ERα-labeling in the VMN, ARC, and PIT, respectively. D1 to D3, Higher-power images illustrating ERα-labeled nuclei from an ERα knockdown (ERα KD) female marmoset that demonstrate greatly diminished ERα-labeling in the VMN and ARC, but with abundant ERα-labeling in PIT. Images illustrated in A1, B1 to B3, and D1 to D3 were obtained from female marmoset ERαKD 1, while those in C1 to C3 were obtained from control marmoset 2. Images of ERα-labeling illustrated in C1 to C3 and D1 to D3 were obtained from adjacent coronal sections approximating the rostral-caudal location illustrated in A1. Scale bar in A, 500 μm. Scale bar in B3, 50 μm, also applies to B1 to D3.

As illustrated by a coronal section from approximately the mid-RC extents of VMN and ARC within the hypothalamus (Fig. 3A1), abundant GFP-ir labeling was demonstrated in VMN and ARC and sometimes in the ventrally adjacent PIT and laterally adjacent optic tracts (Fig. 3A1-3B3). Abundant ERα-ir labeled nuclei within cells of the VMN and ARC were observed in female monkey controls that received hypothalamic infusions of scramble shRNA (Fig. 3C1-3C2), but such ERα protein expression within cells of the VMN and ARC was less pronounced in female monkeys receiving shRNA directed toward *ESR1* (ERαKD in Fig. 3D1-3D2). ERα-ir labeled nuclei within cells in the ventrally adjacent PIT, however, were observed both in control and ERαKD females (Fig. 3C3 and 3D3).

**Figure 4.**
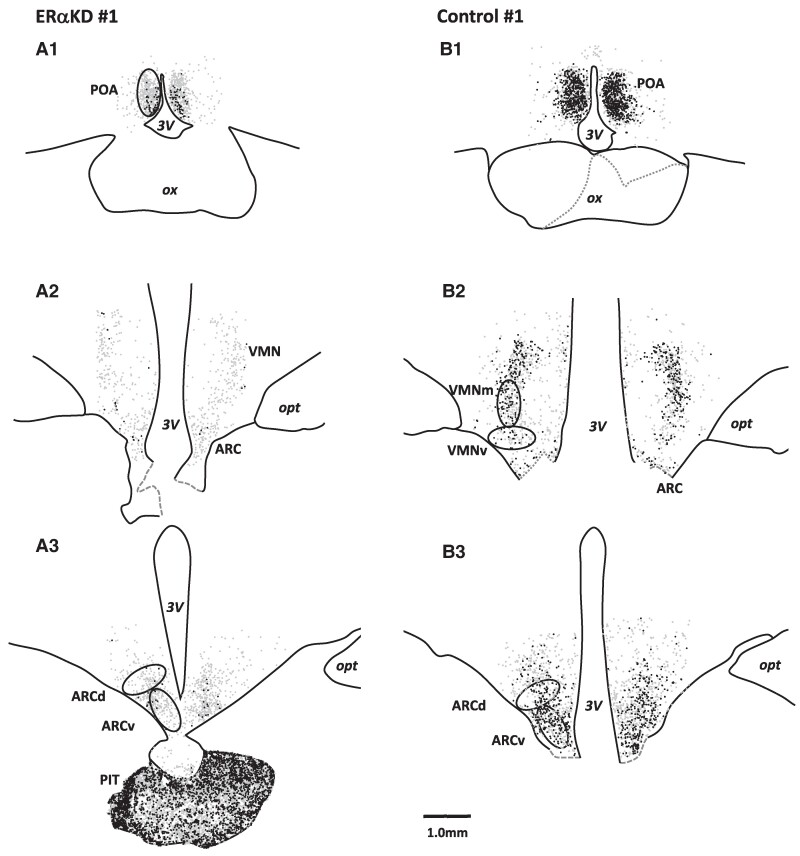
Black and white illustrations derived from microscopic images (×4 magnification) of rostral-caudal series of coronal brain sections representing ERα-labeled neurons in the preoptic area (POA), mid, and ventral aspects of the ventromedial nucleus (VMNm and VMNv, respectively) and dorsal and ventral aspects of the arcuate nucleus (ARCd and ARCv, respectively) in A1 to A3, an ERα knockdown female marmoset, ERαKD 1, and B1 to B3, a control female marmoset, control 1. Ellipses indicate regions of interest for ERα-labeled cell quantification in A1, POA; B2, VMNm and VMNv; and A3 and B3, ARCd and ARCv. Grayscale dots indicate the location of ERα-labeled neurons. Black dots indicate ERα labeled neurons in the upper 50% of labeling intensity range, while light gray dots indicate neurons in the lighter half of the intensity range. A1 and B1 illustrate coronal brain sections through rostral hypothalamus at the level of the optic chiasm (ox), where the highest density of ERα-labeled neurons is found in the POA. A2 and B2 illustrate coronal brain sections through the central rostral-caudal extent of the hypothalamus that contains the VMN. At slightly more caudal locations, as illustrated in B2, we quantified both VMNm and VMNv subregions of the VMN in which the distribution of ERα-labeled neurons extends ventral to the neighboring optic tract (opt). A3 and B3 illustrate coronal brain sections through the caudal hypothalamus where the highest density of ERα-labeled neurons is found in the ARC. We quantified both an ARCd and an ARCv subregion of ARC in which ERα-labeled neurons extended dorso-laterally. B3 also illustrates the anterior pituitary (PIT) robustly expressing ERα-labeled neurons. Representative ellipses (1000× 500 μm) indicate locations from which ERα-label was quantified for each region of interest. 3V, third ventricle.

**Figure 5.**
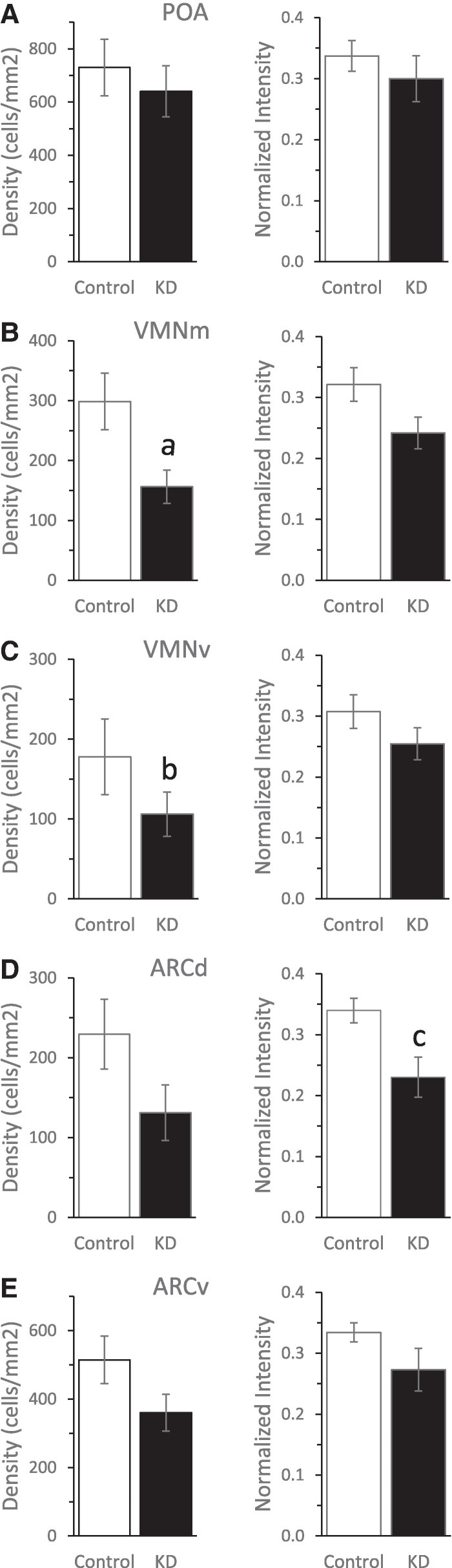
Mean ± SEM values for 5 control (open bars) and 4 ERαKD (filled bars) adult female marmoset monkeys within 5 mediobasal hypothalamic region of interest values with regard to quantified numbers of ERα immunopositive cells per mm^2^ and ERα immunopositive intensity within A, POA; B, VMNm; C, VMNv; D, ARCd; and E, ARCv. Statistically significant differences indicating values in ERαKD female marmosets are less than those of controls: a, *P* = .046; b, *P* = .040; c, *P* = .021. ARCd, dorsal arcuate nucleus; ARCv, ventral arcuate nucleus; POA, preoptic area; VMNm, mid-ventromedial nucleus; VMNv, ventral ventromedial nucleus.

Quantitative assessment of ERα-ir labeled nuclei within cells of the POA, VMN, and ARC ROIs within the mediobasal hypothalamus (Fig. 4A1-4B3) of OVX and midcycle E_2_-replaced adult female marmosets demonstrated clear bilateral KD of ERα protein expression, in terms of cell density or intensity of immunostain, within cells of the targeted VMN and ARC (Fig. 4A2 and 4B2). Intrahypothalamic infusion of AAV8 comprising shRNA specific for *ESR1* into ERαKD female marmosets resulted in 38% to 48% fewer numbers of cells expressing detectable ERα within ROIs incorporating both VMNm and VMNv (Fig. 5B and 5C; *P* = .040 and .046, respectively). In contrast, the ROI incorporating POA exhibited comparable numbers of ERα-immunopositive cells in ERαKD and control females (*P* = .561; Fig. 5A).

Staining intensity of immunopositive ERα cells, however, was diminished by 30% in ERαKD compared to control females within the ROI incorporating the dorsal extent of ARC (ARCd; *P* = .020; Fig. 5D). Nevertheless, when POA, all VMN, and ARCv ROIs were examined for intensity of the immunohistochemical stain for ERα expression, values were similar between the two female groups.

The POA is outside the targeted area, whereas the ARCv is located at its ventral-most limit and neither ROI exhibited statistically significant KD of ERα protein expression (Fig. 5E). Multiple linear regression analyses indicated positive correlations between quantified ERα cell density values in VMN and ARC hypothalamic ROIs alone, VMNm vs VMNv (*P* = .039; *r* = 0.73) and ARCd vs ARCv (*P* = .047; *r* = 0.83), the only ROIs at which viral vector infusions were targeted. Positive correlations were similarly identified between quantified ERα cell intensity values in VMNm vs VMNv (*P* = .001; *r* = 0.94) and ARCd vs ARCv (*P* = .001; *r* = 0.93), but also included POA vs ARCv (*P* = .010; *r* = 0.83).

### Female Sexual Receptivity Diminished by Estrogen Receptor α Knockdown in the Hypothalamic Ventromedial Nucleus and Arcuate Nucleus

**Figure 6.**
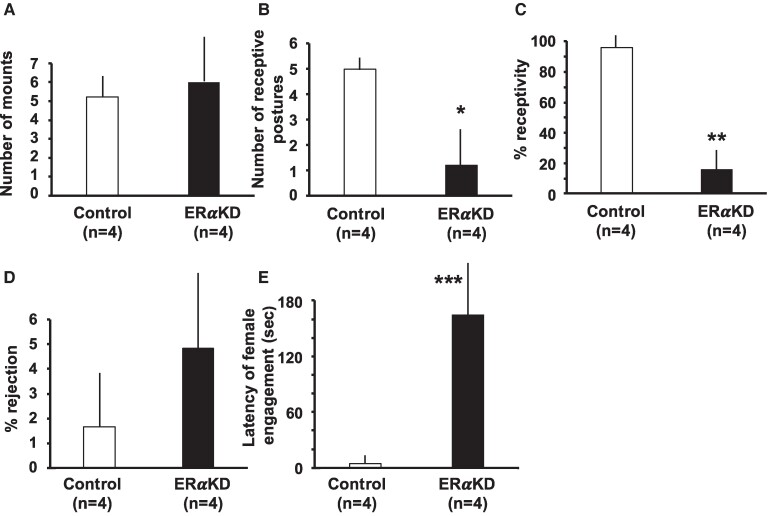
Mean and SEM of sexual behaviors observed in 8 male-female marmoset monkey pairs, 4 comprising control females (open bars) and 4 comprising ERαKD females (filled bars) for A, distance (cm) female marmosets moved their feet away from their hands following a mount by their male partner, in relation to whether intromission occurred; B, time from test onset until female marmosets behaviorally interacted with their male partners, either receptively or in rejection; C, frequencies of male mounting of their female partners; D, total numbers of receptive postures in response to the male mounting; and E, female sexual rejection of their male partners. *P = .03; **P = .01; ***P = .001 vs controls.

Male marmosets appeared equally sexually attracted toward their female partners since frequencies of male mounts did not differ between female groups (Fig. 6A). In contrast to comparability of male sexual interest in female marmosets from both treatment groups, KD of ERα in the VMN and ARC of female marmosets extinguished more than 80% of female receptive postures (*P* = .03), and thus female sexual receptivity (*P* = .01) (Fig. 6B and 6C). Such a dramatic loss of receptivity to male sexual advances among ERαKD females was due to their failing to adopt a receptive posture on a vertical surface during 37% of male mounts (distance feet moved away from hands, 0.1 ± 0.1 cm). All movements were less than 0.5 cm and thus insufficient to enable male intromission (distance hands moved away from feet when enabling intromission: control females, 1.6 ± 0.5 cm; ERαKD females, 1.8 ± 0.9 cm). Control females, in contrast, terminated mounts by moving away from the male. ERαKD females, nevertheless, did not increase their percentage of sexual rejection toward male partners (Fig. 6D). Interestingly, when ERαKD females did exhibit the occasional receptive or rejection sexual response to a male, the latencies to such responses following the onset of a male mount were atypically tardy (*P* = .001) for female marmosets (Fig. 6E).

In addition, we found statistically significant correlations between the number of ERα-immunopositive cells and the frequency of female marmoset sexual behavior, but within the VMNm alone (Table 2). The number of ERα-immunopositive cells in the VMN positively correlated with female sexually receptive postures in response to male mounts (*P* = .032), percentage of female receptivity (*P* = .037), and latency of the female to respond to a male mount (*P* = .041).

**Table 2.** Multiple linear regression correlations involving female sexual behavior parameters, circulating gonadotropin levels, and metabolic assessments that yielded statistically significant differences between female groups and neuroimmunohistochemical quantification of mediobasal hypothalamic expression of estrogen receptor α cell density immunostaining

Parameter	ERα cell density quantification				
Female sexual behavior*^a^*	ROI	*P*	Slope	*r*	% Variance explained
Receptive posture (No./30 min)	**VMNm**	**.032**	+0.02	**0.75**	56
% Receptivity, % of tests	**VMNm**	**.037**	+0.28	**0.74**	54
Latency to respond, s	**VMNm**	**.041**	−0.01	**0.73**	53
**Gonadotropin levels**					
Change in CG levels from baseline, ng/mL	None	.562	—	0.24	6
**Metabolic assessment**					
AUC glucose during OGTT, mg/dL × min	None	.105	—	0.61	38
AUC calories consumed, kcal × wk	**VMNv**	**.003**	+1.62	**0.93**	86
AUC calories consumed, kcal × wk	**ARCv**	**.008**	−0.39	**0.93**	86

Abbreviations: ARCv, ventral arcuate nucleus; AUC, area under the curve; CG, chorionic gonadotropin, marmoset equivalent of luteinizing hormone released from anterior pituitary; ERα, estrogen receptor α; OGTT, oral glucose tolerance test; ROI, region of interest; VMNm, midventromedial nucleus.

^*a*^Mean values for each monkey across all behavior tests; bold values indicate statistically significant correlations (*P* < .05).

**Figure 7.**
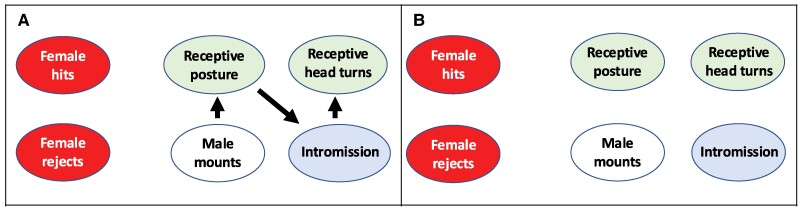
Species typical sexual behavior is observed in the behavioral transitions most likely to occur with A, control females. In contrast, B, loss of estradiol action within the ventromedial nucleus (VMN) and arcuate nucleus (ARC) of the hypothalamus of ERαKD female marmosets illustrates the switch in likelihood of behavioral transition from sexual receptivity to absence of female sexual receptivity and rejection. Each black arrow represents a statistically significant (*P* < .05) transition between connected behaviors. Green circles indicate sexually receptive behavior, blue circles indicate male intromission, and red circles indicate sexual rejection.

Behavioral sequence analyses revealed ERαKD-mediated disruption to marmoset-typical behavioral transitions within male-female sexual interactions as illustrated by frequency distributions for each group and analyzed transitions in Table 3. Frequencies in parentheses are the expected frequencies within the entire cohort of 11 male-female pairs observed. Frequencies not in parentheses depict the observed frequencies of a specified behavioral transition, such as male mount female–female accepts male mount for each treatment group. Fig. 7A illustrates a marmoset-typical sexual behavior pattern between male and female pairmates in the control group constructed from chi-square values for each behavioral transition and their derived *P* values from Table 3. In this figure, the most likely behavioral sequence following male initiation of a mount is male mount followed by female receptive posture (*P* < .001). Subsequent transitions then follow, resulting in a transition from female receptive posture to male intromission (*P* < .0001), followed by male intromission leading to female receptive head turn (*P* < .0001). Fig. 7B subsequently illustrates the behavioral sequence consequences of ERαKD in the hypothalamic VMN. Such hypothalamic ERαKD, alone, is sufficient to abolish all behavioral transitions essential to female marmoset-typical sexual behavior.

**Table 3.** A, Frequency distribution table illustrates the observed frequencies of subsequent behaviors following initial behaviors (values outside parentheses) compared to expected frequencies of these behavioral transitions for all male-female pair interactions during behavioral tests, and B, subtables B.1 to B.3 show the χ^2^ statistic for all behavioral transitions analyzed for each female group

A, Frequency distribution table							
		Subsequent behavior					
		Treatment group	RP	RJ	RHT	I	H
Initial behavior	M	Control (scramble)	2.27 (0.78)	0.09 (0.48)	—	—	—
		ERα knockdown	0.57 (0.78)	0.19 (0.48)	—	—	—
	RP	Control (scramble)	—	—	2.09 (0.52)	2.27 (0.57)	—
		ERα knockdown	—	—	0.38 (0.52)	0.57 (0.57)	—
	RJ	Control (scramble)	—	—	—	—	0 (0.20)
		ERα knockdown	—	—	—	—	0 (0.20)

B, *x^2^* statistic for all behavioral transitions analyzed	
B.1. Behavioral transition	Treatment group: control
M->RP	χ^2^: 28.57; *P* < .001
M->RJ	χ^2^: 3.20; ns
RP->RHT	χ^2^: 59.05; *P* < .0001
RP->I	χ^2^: 50.86; *P* < .0001
RJ->H	N/A

B.2. Behavioral transition	Treatment group: ERα knockdown
M->RP	χ^2^: 0.56; ns
M->RJ	χ^2^: 1.75; ns
RP->RHT	χ^2^: 0.05; ns
RP->I	χ^2^: 0.00004; ns
RJ->H	N/A

These data are represented graphically in the behavioral transitions diagram in Fig. 7.

Abbreviations: M, male mount; ERα, estrogen receptor α; H, hitting partner; I, penile intromission; N/A, absence of occurrence; ns, not significant; RHT, sexually receptive head turn; RJ, sexual rejection behavior; RP, sexually receptive behavior.

### Diminished Estradiol-mediated Negative Feedback on Circulating Levels of Pituitary Chorionic Gonadotropin Following Estrogen Receptor α Knockdown in the Mediobasal Hypothalamus

**Figure 8.**
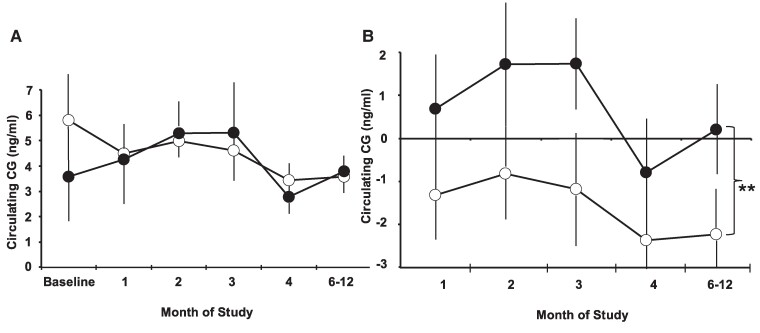
Mean and SEM, circulating levels of pituitary luteinizing hormone–like chorionic gonadotropin (CG) in 5 control (open symbols) and 5 ERαKD female (filled symbols) marmoset monkeys during months 1 to 12 following study onset: A, circulating levels of CG (ng/mL), and B, changes in circulating CG levels relative to baseline (ng/mL), **P = .002 ERαKD vs controls, all time points combined.

Since the numbers of SC-implanted, E_2_-filled Silastic capsules were increased to mid-cycle E_2_-levels only approximately 1 month before study onset, our expectations were for diminishing circulating CG levels in control females and increased or comparatively greater circulating CG levels in ERαKD females during the study. Consistent with this expectation, modest changes in CG levels from baseline (*P* = .002) were greater in ERαKD compared to control females (Fig. 8B), particularly during 1 to 3 months following study onset. Mean CG levels (*P* = .684) were, however, comparable between female groups (Fig. 8A). Circulating E_2_ levels during study months 6 to 12 were typical of mid-cycle levels and comparable between treatment groups (Table 4). Androgens, progestins, and glucocorticoids were also comparable between treatment groups. There was no statistically significant correlation between changes in circulating CG levels and ERα expression in POA, VMN, and ARC nuclei of the marmoset hypothalamus (see Table 2).

**Table 4.** Circulating hormone levels (mean ± SEM) in control (n = 6) and estrogen receptor α knockdown (n = 5) adult female marmoset monkeys (ovariectomized and implanted subcutaneously with estradiol capsules) during 6 to 12 months following study onset

Hormone	Control (n = 6)	ERαKD (n = 5)	*P*
Estradiol, pg/mL	895 ± 62	815 ± 82	.59
Estrone, pg/mL	175 ± 20	192 ± 32	.83
Testosterone, ng/mL	0.3 ± 0.1	0.5 ± 0.2	.33
Androstenedione, ng/mL	2.5 ± 0.4	2.7 ± 0.5	.98
DHEA, ng/mL	2.7 ± 0.5	2.0 ± 0.5	.62
17-OHP, ng/mL	7.4 ± 1.1	7.6 ± 1.4	.78
Progesterone, ng/mL	2.5 ± 1.0	2.8 ± 1.0	.89
Cortisol, ng/mL	823 ± 113	884 ± 61	.97
Cortisone, ng/mL	600 ± 44	486 ± 124	.36
Aldosterone, ng/mL	596 ± 42	479 ± 132	.43

Abbreviations: 17-OHP, 17-hydroxyprogesterone; DHEA, dehydroepiandrosterone; ERαKD, estrogen receptor α knockdown.

### Subtle Decline in Calorie Consumption Accompanying Diet-induced Obesity–induced Increases in Body Weight and Fat Mass Following Estrogen Receptor α Knockdown in the Mediobasal Hypothalamus

**Figure 9.**
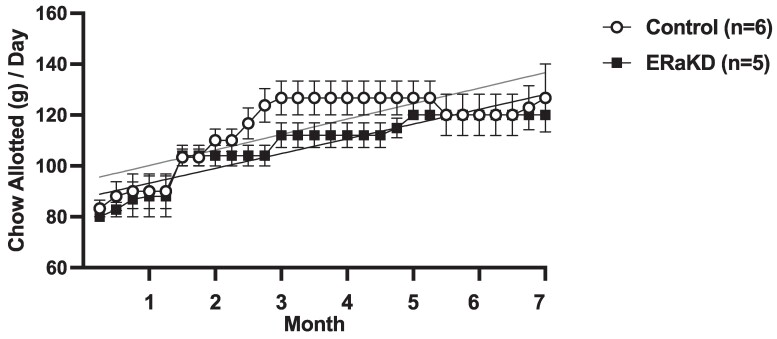
Mean and SEM weekly chow allotments provided to 6 male-female pairs from the control female group (open symbols) and 5 male-female pairs from the ERαKD female group (filled symbols) illustrating progressive, parallel increases in chow amounts until month 7 of the study in both female groups. Slopes indicate comparable trajectories of progressive increases in chow allotments to control (gray line: slope = 1.5, *r* = 0.57) and ERαKD (black line: slope = 1.4, *r* = 0.76) female groups. After 7 months of study, females were housed separately from their male pairmates during study months 8 to 9 to permit individual calorie consumption evaluation.

Comparable amounts of chow per day, and their weekly increments, were allotted to control and ERαKD females and their male pairmates when housed together (Fig. 9). When control and ERαKD females were later housed singly during study months 8 and 9, chow allotments diminished to 50% of those provided when females were housed with their male pairmates, but remained comparable between female groups (control, 66 ± 2 g/day; ERαKD, 66 ± 2 g/day).

**Figure 10.**
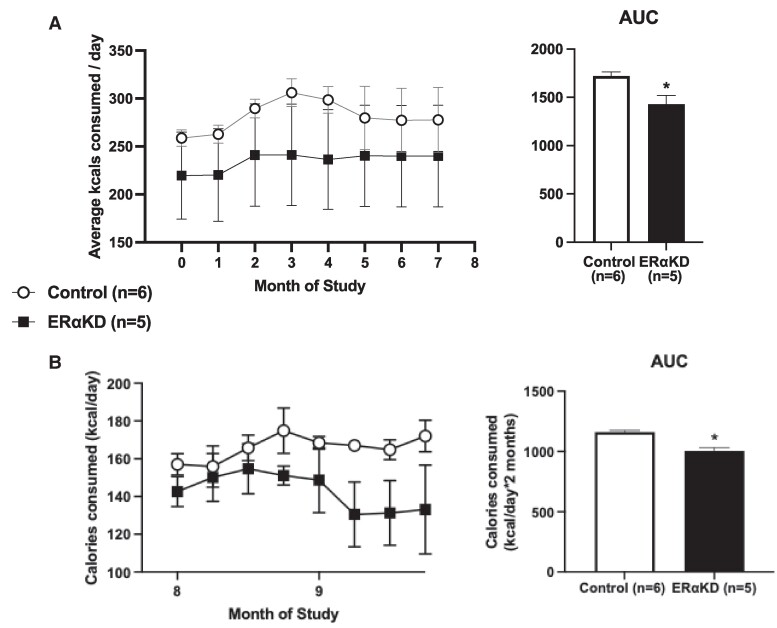
Mean and SEM calories consumed by A, male-female pairs during study months 1 to 7 comprising 6 female pairmates from the control group (open filled symbols and bars) and 5 from the ERαKD group (filled symbols and bars) as well as area under the curve (AUC) calories consumed by the pairs for the entire 7-month period, and by B, singly housed females comprising 6 from the control female group (open symbols and bars) and 5 from the ERαKD female group (filled symbols and bars) during months 8 to 9 following study onset as well as AUC calories consumed by females alone. *P = .001 vs controls. Y-axes for calories consumed per day in panels A and B commence at 150 and 100, respectively, to better appreciate consistent lower calorie consumption by the ERαKD female group.

Male-female pairs from the ERαKD female group consumed fewer calories (*P* = .02) than control male-female pairs during weekly increments of daily calories from months 1 to 7 following OVX (Fig. 10A). When females were separated from their male pairmates at 8 to 9 months following OVX for individual behavioral and calorie intake assessments, total daily calories provided to both female groups were comparable, with singly housed females from both groups consuming approximately 80% of calories provided (see Fig. 10). On examining calories consumed when females were housed singly, ERαKD females consumed (*P* = .001) fewer calories (AUC kcal corrected for FFM, 1004 ± 12 kcal/g FFM × 56 days) in comparison to control females (1161 ± 6 kcal/g FFM × 56 days). The number of ERα-immunopositive cells (density) in the VMNv and ARCv correlated positively and negatively, respectively, with calorie consumption (see Table 2). We did not, however, achieve statistically significant KD of ERα in the ARCv. No correlations were found between hypothalamic ERα-immunopositive parameters and AUC glucose levels during an OGTT (see Table 2).

**Figure 11.**
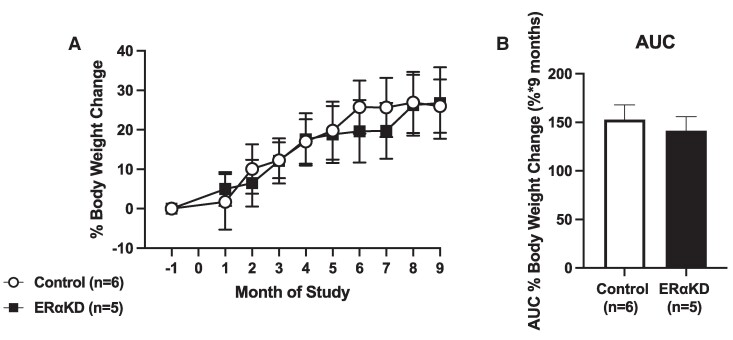
Comparable mean and SEM A, percentage of female body weight change from baseline, and B, area under the curve percentage female body weight mass during study months −1 to 9 in 6 control (open symbols and bars) and 5 ERαKD (filled symbols and bars) female marmoset monkeys.

On examining parameters assessing body mass and body composition parameters, ERαKD females exhibited comparable increases to control females both in body weight and fat mass (Table 5) and in percentage of body weight gained (Fig. 11A and 11B) induced by DIO. There were no obvious between female group changes in other DXA-determined body composition parameters at 3 or 6 months following ERα KD, including abdomen and hips/thighs (see Table 5). DXA-determined BMC and BMD also failed to differ between female groups between baseline and 3 or 6 months following ERα KD, including BMC and BMD in previously validated body ROIs such as the abdomen, comprising mostly the lumber spine (see Table 5).

**Table 5.** Regional body composition parameters (mean ± SEM) as determined by dual-energy x-ray absorptiometry in control (n = 6) and estrogen receptor α knockdown (n = 5) adult female marmoset monkeys (ovariectomized and implanted subcutaneously with estradiol capsules) at baseline, 3, and 6 months following study onset, changes, and percentage changes from baseline at 3 and 6 months

Body region of interest	DXA parameter	Treatment group	Baseline	3 mo	6 mo	Change, baseline to 3 mo	Change baseline to 6 mo	% Change, baseline to 3 mo	% Change, baseline to 6 mo
Total body	**Body weight, g**	Control	422.5 ± 19.2	472.4 ± 23.0	525.7 ± 16.6	57.8 ± 21.4	102.2 ± 21.0	14.5 ± 5.1	26.1 ± 7.0
		ERαKD	421.8 ± 30.2	467.5 ± 20.9	496.9 ± 20.1	43.0 ± 17.2	73.2 ± 25.1	11.9 ± 23.3	20.0 ± 25.8
		**Groups combined** *^a^*	**378.5 ± 15.5**	**429.5 ± 15.0**	**467.3 ± 13.2**	—	—	—	—
	**Fat mass, g**	Control	107.4 ± 15.6	174.3 ± 13.0	199.5 ± 15.9	64.0 ± 22.9	49.4 ± 30.9	78.7 ± 33.5	114.1 ± 65.5
		ERαKD	113.5 ± 20.6	161.4 ± 20.6	182.2 ± 22.1	49.6 ± 27.8	63.6 ± 33.4	62.6 ± 35.1	86.8 ± 42.7
		**Groups combined** *^b^*	**110.7 ± 12.7**	**168.5 ± 11.3**	**191.6 ± 12.9**	—	—	—	—
	FFM, g	Control	271.8 ± 7.9	261.0 ± 17.7	280.5 ± 15.8	−3.6 ± 14.0	18.6 ± 13.8	−1.7 ± 5.1	5.0 ± 5.2
		ERαKD	264.5 ± 9.4	261.2 ± 23.0	269.8 ± 20.6	1.2 ± 13.4	14.2 ± 17.1	−1.0 ± 4.8	3.0 ± 5.7
	Fat/FFM ratio	Control	0.4 ± 0.01	0.7 ± 0.1	0.7 ± 0.1	0.3 ± 0.1	0.3 ± 0.2	87.5 ± 43.3	116.5 ± 80.3
		ERαKD	0.4 ± 0.1	0.6 ± 0.1	0.7 ± 0.1	0.2 ± 0.2	0.3 ± 0.2	73.3 ± 50.5	90.4 ± 54.4
	% Fat	Control	25.8 ± 3.0	37.6 ± 2.5	38.4 ± 2.6	10.9 ± 4.5	5.5 ± 5.7	53.6 ± 26.0	61.9 ± 39.1
		ERαKD	27.0 ± 3.1	35.9 ± 4.3	37.8 ± 3.9	8.7 ± 5.6	9.2 ± 5.8	40.6 ± 23.0	49.1 ± 23.4
	% FFM	Control	66.9 ± 2.7	56.1 ± 2.0	54.2 ± 3.0	−9.5 ± 3.9	−5.2 ± 5.3	−13.5 ± 5.5	−15.4 ± 6.8
		ERαKD	65.8 ± 3.0	58.1 ± 4.2	56.3 ± 3.7	−8.0 ± 5.2	−8.8 ± 5.8	−10.1 ± 7.2	12.2 ± 8.1
	BMC, g	Control	15.1 ± 2.9	15.7 ± 0.9	16.8 ± 0.5	0.5 ± 0.6	1.2 ± 0.5	3.4 ± 4.5	11.9 ± 4.4
		ERαKD	14.4 ± 0.6	15.0 ± 0.5	15.4 ± 0.5	0.9 ± 0.7	1.8 ± 0.7	6.0 ± 4.7	9.6 ± 5.4
	BMD, g/cm^2^	Control	0.2 ± 0.1	0.2 ± 0.1	0.2 ± 0.1	0.01 ± 0.01	0.01 ± 0.01	1.7 ± 4.5	6.0 ± 1.6
		ERαKD	0.2 ± 0.1	0.2 ± 0.01	0.2 ± 0.01	0.01 ± 0.01	0.01 ± 0.01	1.6 ± 3.7	3.6 ± 4.1
Abdomen	Fat, g	Control	22.6 ± 4.3	40.8 ± 3.1	53.0 ± 7.1	16.6 ± 6.4	26.8 ± 11.4	119.5 ± 65.0	213.5 ± 142.8
		ERαKD	23.5 ± 5.5	38.6 ± 6.9	45.0 ± 7.9	14.0 ± 8.2	20.4 ± 10.4	101.2 ± 66.6	143.5 ± 87.1
	FFM, g	Control	80.2 ± 1.7	92.8 ± 8.3	109.0 ± 7.4	14.0 ± 11.1	28.4 ± 9.8	18.3 ± 14.5	36.1 ± 12.9
		ERαKD	89.8 ± 5.8	94.6 ± 9.5	106.4 ± 10.0	4.6 ± 4.4	16.4 ± 8.3	4.6 ± 4.5	18.8 ± 8.3
	Fat/FFM ratio	Control	0.3 ± 0.1	0.5 ± 0.1	0.5 ± 0.1	0.1 ± 0.1	0.2 ± 0.2	109.7 ± 84.2	169.9 ± 152.4
		ERαKD	0.3 ± 0.1	0.4 ± 0.1	0.4 ± 0.1	0.3 ± 0.1	0.2 ± 0.1	99.3 ± 70.8	113.2 ± 77.7
	% Fat	Control	21.4 ± 3.4	30.0 ± 2.7	32.6 ± 4.0	8.5 ± 5.6	9.9 ± 7.6	69.4 ± 52.7	89.2 ± 76.5
		ERαKD	19.8 ± 3.2	28.9 ± 5.0	29.5 ± 4.7	8.6 ± 6.0	9.2 ± 6.6	59.7 ± 39.5	67.4 ± 41.9
	% FFM	Control	78.6 ± 3.4	69.0 ± 2.7	67.4 ± 4.0	−8.5 ± 5.6	−9.9 ± 7.6	−9.8 ± 6.6	−11.2 ± 8.5
		ERαKD	80.2 ± 3.2	71.0 ± 5.0	70.5 ± 4.7	−8.6 ± 6.0	−9.2 ± 6.6	−10.0 ± 7.3	−10.3 ± 8.1
	BMC, g	Control	2.1 ± 0.12	2.4 ± 0.14	2.6 ± 0.12	0.3 ± 0.1	0.5 ± 0.1	12.1 ± 3.7	23.9 ± 4.1
		ERαKD	2.1 ± 0.13	2.3 ± 0.14	2.4 ± 0.14	0.2 ± 0.1	0.3 ± 0.1	10.6 ± 6.0	17.4 ± 5.7
	BMD, g/cm^2^	Control	0.16 ± 0.01	0.16 ± 0.01	0.16 ± 0.01	0.003 ± 0.003	0.003 ± 0.003	1.9 ± 2.2	2.1 ± 1.9
		ERαKD	0.16 ± 0.01	0.15 ± 0.01	0.16 ± 0.01	0.003 ± 0.004	0.003 ± 0.004	1.8 ± 3.2	2.3 ± 2.5
Chest	Fat, g	Control	35.6 ± 6.6	60.7 ± 5.0	70.3 ± 5.1	25.0 ± 8.2	33.0 ± 10.9	114.8 ± 60.6	176.0 ± 118.3
		ERαKD	39.7 ± 9.0	53.2 ± 6.5	61.8 ± 7.0	14.2 ± 10.0	22.8 ± 12.6	81.4 ± 55.8	118.9 ± 74.2
	FFM, g	Control	62.0 ± 2.3	50.8 ± 4.1	48.7 ± 4.4	−8.2 ± 2.5	−11.4 ± 3.1	−13.3 ± 4.1	−18.9 ± 5.5
		ERαKD	56.7 ± 2.8	52.8 ± 6.9	49.8 ± 3.7	−3.8 ± 5.6	−6.8 ± 4.1	−7.1 ± 10.0	−11.2 ± 7.6
	Fat/FFM ratio	Control	0.6 ± 0.1	1.2 ± 0.1	1.5 ± 0.2	0.6 ± 0.2	0.9 ± 0.3	159.9 ± 84.6	290.9 ± 208.1
		ERαKD	0.7 ± 0.2	1.1 ± 0.2	1.3 ± 0.3	0.4 ± 0.3	0.6 ± 0.4	127.7 ± 92.8	174.8 ± 111.8
	% Fat	Control	35.1 ± 5.3	54.3 ± 2.6	59.1 ± 3.4	17.6 ± 6.2	22.4 ± 8.0	74.1 ± 40.7	98.4 ± 61.2
		ERαKD	39.0 ± 5.6	50.3 ± 5.5	54.9 ± 4.1	12.1 ± 8.5	16.7 ± 8.5	50.7 ± 33.7	65.1 ± 34.2
	% FFM	Control	64.9 ± 5.3	45.7 ± 2.6	40.9 ± 3.4	−17.6 ± 6.2	−22.4 ± 8.0	−24.9 ± 7.9	−32.1 ± 8.3
		ERαKD	60.0 ± 5.6	49.7 ± 5.5	45.1 ± 4.1	−12.1 ± 8.5	−16.7 ± 8.5	−15.2 ± 12.2	−21.9 ± 12.2
	BMC, g	Control	2.9 ± 0.17	3.1 ± 0.17	3.3 ± 0.15	0.3 ± 0.2	0.4 ± 0.3	9.8 ± 6.9	14.5 ± 11.0
		ERαKD	2.6 ± 0.19	2.8 ± 0.10	2.9 ± 0.18	0.2 ± 0.3	0.2 ± 0.3	10.7 ± 10.5	13.2 ± 14.5
	BMD, g/cm^2^	Control	0.14 ± 0.002	0.14 ± 0.003	0.15 ± 0.003	0.001 ± 0.004	0.01 ± 0.01	1.3 ± 3.2	6.8 ± 5.0
		ERαKD	0.13 ± 0.01	0.14 ± 0.01	0.14 ± 0.003	0.01 ± 0.01	0.01 ± 0.01	9.7 ± 6.5	10.2 ± 7.2
Upper legs	Fat, g	Control	10.2 ± 0.9	16.5 ± 2.2	17.5 ± 1.4	7.0 ± 2.6	7.8 ± 2.2	73.0 ± 25.4	87.0 ± 34.1
		ERαKD	10.0 ± 0.8	16.2 ± 2.4	16.2 ± 2.7	6.2 ± 2.4	6.2 ± 2.9	66.3 ± 27.2	68.4 ± 32.0
	FFM, g	Control	50.6 ± 2.9	46.0 ± 3.0	49.3 ± 3.0	−2.9 ± 0.7	1.0 ± 0.9	−5.1 ± 1.2	2.3 ± 1.8
		ERαKD	44.2 ± 2.2	44.4 ± 2.1	42.2 ± 1.5	1.2 ± 3.4	−1.0 ± 2.4	4.2 ± 8.3	−1.4 ± 5.5
	Fat/FFM ratio	Control	0.2 ± 0.003	0.4 ± 0.1	0.4 ± 0.003	0.2 ± 0.1	0.2 ± 0.1	82.9 ± 27.3	82.7 ± 32.4
		ERαKD	0.2 ± 0.0	0.4 ± 0.1	0.4 ± 0.1	0.1 ± 0.1	0.2 ± 0.1	65.7 ± 33.3	73.9 ± 34.8
	% Fat	Control	16.7 ± 1.1	26.2 ± 2.5	26.2 ± 1.7	9.4 ± 3.2	9.1 ± 2.8	58.2 ± 18.2	58.9 ± 21.3
		ERαKD	18.5 ± 1.4	26.6 ± 3.8	27.3 ± 3.6	7.8 ± 3.4	8.5 ± 4.1	43.8 ± 21.1	49.2 ± 21.4
	% FFM	Control	83.3 ± 1.1	73.8 ± 2.5	73.8 ± 1.7	−9.4 ± 3.2	−9.1 ± 2.8	−11.2 ± 3.9	−10.7 ± 3.2
		ERαKD	81.5 ± 1.4	73.4 ± 3.8	72.8 ± 1.7	−7.8 ± 3.4	−8.5 ± 4.1	−9.5 ± 4.7	−10.2 ± 5.1
	BMC, g	Control	1.6 ± 0.23	1.8 ± 0.10	2.0 ± 0.11	0.3 ± 0.1	0.4 ± 0.2	27.2 ± 18.3	38.3 ± 22.3
		ERαKD	1.7 ± 0.07	1.7 ± 0.06	1.7 ± 0.02	0.1 ± 0.1	0.1 ± 0.1	4.4 ± 4.7	6.2 ± 6.6
	BMD, g/cm^2^	Control	0.16 ± 0.01	0.17 ± 0.002	0.17 ± 0.002	0.004 ± 0.004	0.01 ± 0.004	2.4 ± 2.1	5.6 ± 2.3
		ERαKD	0.16 ± 0.01	0.16 ± 0.01	0.17 ± 0.01	0.01 ± 0.003	0.01 ± 0.003	3.7 ± 1.9	7.5 ± 1.9
Extremities	Fat, g	Control	23.8 ± 2.5	34.2 ± 3.4	33.8 ± 2.4	10.4 ± 4.1	10.0 ± 3.9	50.6 ± 20.2	51.3 ± 24.8
(lower legs + arms)		ERαKD	25.5 ± 5.8	32.4 ± 3.8	36.0 ± 3.9	5.8 ± 5.2	8.8 ± 5.9	9.6 ± 6.6	58.9 ± 24.5
	FFM, g	Control	54.4 ± 2.1	50.8 ± 5.5	53.8 ± 2.3	−3.6 ± 3.8	−1.7 ± 0.9	−7.4 ± 7.2	−1.1 ± 1.8
		ERαKD	50.3 ± 4.2	47.8 ± 4.0	51.2 ± 5.9	−1.7 ± 0.9	1.2 ± 1.9	−4.0 ± 1.9	1.9 ± 3.4
	Fat/FFM ratio	Control	0.4 ± 0.04	0.7 ± 0.1	0.7 ± 0.1	0.3 ± 0.1	0.2 ± 0.1	82.9 ± 27.3	82.7 ± 32.4
		ERαKD	0.5 ± 0.1	0.7 ± 0.1	0.7 ± 0.1	0.2 ± 0.1	0.3 ± 0.2	65.7 ± 33.3	73.9 ± 34.8
	% Fat	Control	60.5 ± 4.0	82.0 ± 9.2	77.2 ± 3.2	21.5 ± 9.2	16.7 ± 6.5	37.4 ± 15.7	31.1 ± 14.1
		ERαKD	65.4 ± 5.8	80.6 ± 7.2	83.1 ± 7.6	12.7 ± 8.0	14.8 ± 9.5	26.0 ± 12.3	30.6 ± 13.7
	% FFM	Control	139.5 ± 4.0	118.0 ± 9.2	122.8 ± 3.2	−21.5 ± 9.2	−16.7 ± 6.5	−15.3 ± 6.8	−11.6 ± 4.2
		ERαKD	134.6 ± 5.8	119.4 ± 7.2	116.9 ± 7.6	−12.7 ± 8.0	−14.8 ± 9.5	−10.9 ± 6.1	−12.5 ± 7.4
	BMC, g	Control	4.7 ± 0.22	4.4 ± 0.49	4.9 ± 0.18	−0.34 ± 0.5	0.2 ± 0.1	−7.4 ± 11.3	4.7 ± 3.0
		ERαKD	4.4 ± 0.20	4.8 ± 0.13	4.7 ± 0.14	0.1 ± 0.2	0.3 ± 0.2	2.4 ± 4.2	6.9 ± 4.1
	BMD, g/cm^2^	Control	0.26 ± 0.01	0.27 ± 0.01	0.27 ± 0.003	0.01 ± 0.01	0.01 ± 0.01	2.8 ± 4.0	3.8 ± 1.9
		ERαKD	0.26 ± 0.01	0.30 ± 0.03	0.27 ± 0.01	0.04 ± 0.03	0.01 ± 0.01	16.3 ± 11.1	5.7 ± 3.5
Trunk/Extremities ratio	Fat	Control	2.3 ± 0.3	3.0 ± 0.2	3.5 ± 0.3	0.6 ± 0.3	1.1 ± 0.5	5.7 ± 6.7	11.9 ± 8.0
(trunk = abdomen + chest)		ERαKD	2.4 ± 0.3	2.8 ± 0.1	2.9 ± 0.2	0.4 ± 0.3	0.5 ± 0.3	57.5 ± 20.5	86.4 ± 39.0
Abdomen/Upper legs ratio	Fat	Control	2.1 ± 0.3	2.4 ± 0.2	2.7 ± 0.3	0.3 ± 0.3	0.6 ± 0.4	−4.7 ± 10.6	14.2 ± 8.4
		ERαKD	2.2 ± 0.4	2.6 ± 0.2	2.8 ± 0.3	0.03 ± 0.4	0.5 ± 0.3	35.0 ± 25.3	62.3 ± 32.3

Statistically significant differences across time for increases in body composition parameters (shown in bold) have been generated by the time effect factor in the mixed-models analysis of variance.

Abbreviations: BMC, bone mineral content; BMD, bone mineral density; DXA, dual-energy x-ray absorptiometry; ERαKD, estrogen receptor α knockdown; FFM, fat free mass.

^*a*^ *P* equals .001.

^*b*^ *P* equals .01.

### Locomotor Activity Is Unchanged Following Estrogen Receptor α Knockdown in the Mediobasal Hypothalamus

**Figure 12.**
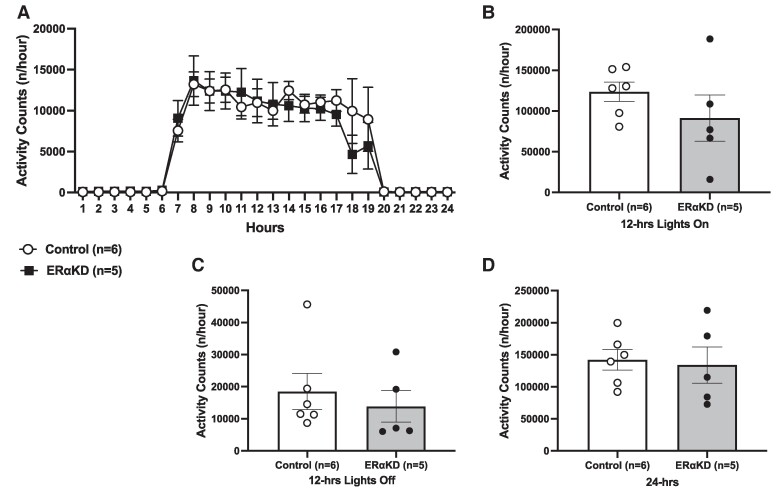
Comparable mean and SEM of A, hourly frequencies for locomotor activity exhibited by 6 control (open symbols and bars) and 5 ERαKD (filled symbols and bars) female marmoset monkeys during the 24-hour light-dark period across a 21-day period during study month 6, in addition to area under the curve of frequencies of locomotor activity during B, 12 hours daytime (*P* = .292; between female groups); C, 12 hours nighttime (*P* = .560; between female groups); and D, the 24-hour time period (*P* = .798; between female groups).

Activity collar assessments of female locomotion were obtained at 6 months post AAV8 intrahypothalamic infusion (while pair-housed with their male cagemate). AUC locomotor activity was not different during the daytime in ERαKD compared to control females (Fig. 12A and 12B). Likewise, during the resting hours of nighttime, there were no differences in locomotor activity between female groups (see Fig. 12A and 12C).

### Fasting Glucose

**Figure 13.**
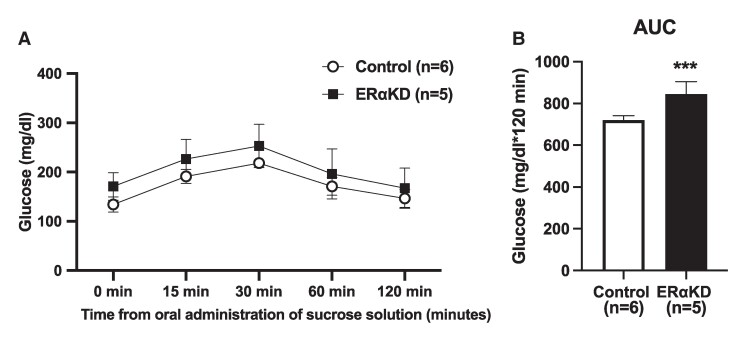
Mean and SEM of A, circulating glucose levels during a 120-minute oral glucose tolerance test (OGTT) administered immediately following 0 minutes, and B, area under the curve (AUC) glucose during the OGTT in 6 control (open symbols and bars) and 5 ERαKD (filled symbols and bars) female marmoset monkeys. AUC glucose was elevated (*P = .001) in ERαKD compared to control females.

Fasting glucose levels remained comparable across female groups at baseline and at 6 months following ERα KD, as was 2-hour glucose derived from an OGTT performed at 6 months following ERα knockdown (Fig. 13A). AUC glucose derived from the 6-month OGTT, however, revealed hyperglycemia in ERαKD females, alone (*P* = .001) (Fig. 13B). No correlations were found between AUC glucose and hypothalamic ERα-immunopositive parameters (see Table 3).

## Discussion

Hypothalamic neuronal receptor mechanisms governing E_2_ regulation of female primate sexual behavior, reproductive hormone secretions, and metabolic function were previously unknown. In the present study, we used an ERα gene KD method to demonstrate that diminished ERα expression in the mediobasal hypothalamus of the OVX, E_2_-replaced marmoset monkey results in a major reduction in female sexual receptivity. These findings provide the first evidence implicating activation of ERα as a key hypothalamic mechanism for E_2_ regulation of female sexual receptivity in an NHP, and thus implicate a similar role for the receptor in other female NHPs, as well as in women. The hypothalamic ERα KD achieved in this study, however, resulted in relatively minor observable effect on gonadotropin secretion and metabolic function. Thus, while our findings clearly reveal a role for mediobasal hypothalamic ERα in female sexual motivation in a primate, the potential involvement of these receptors in negative feedback control in the female reproductive axis, and in E_2_ regulation of female energy homeostasis, will require further clarification.

The present study was partly modeled after published gene-silencing approaches examining ERα's physiological roles in discrete nuclei of the hypothalamus in female rats [32]. In those studies, ERα gene silencing of more than 80% in the VMN abolished female sexual proceptivity and receptivity, attenuated progesterone receptor expression [32], and induced metabolic syndrome [45]. Compared with approaches employed with female rats, we employed MRI guidance to refine individual targeting of the VMN, as NHPs have notable individual variations in neuroanatomical locations [76]. Based on immunohistochemistry analysis of ERα expression in marmoset MBH, ERα expression was significantly diminished in the VMN of females receiving ERα silencing shRNA, as indicated by a 40% to 48% reduction in the density of detectable ERα-expressing cells, while the intensity of ERα expression in the ARC was also diminished by approximately 24% to 30%, with an absence of significantly diminished ERα-expression in the POA. While these percentage reductions of ERα expression achieved in the marmoset did not reach the extent produced by the gene-silencing approach in rodents, they were nevertheless accompanied by major reductions in female sexual receptivity. Our findings reveal that a moderate reduction in ERα expression in the VMN and ARC nucleus is sufficient to virtually abolish female sexual receptivity in E_2_-replaced OVX marmosets, and to significantly prolong the latency for males to mount their female partners. On the basis of these results, we speculate that the maintenance of species-typical female sexual behavior requires the integrated activity of a network of ERα-expressing neurons that are relatively intolerant of the inactivation of components of the network. We also observed a positive correlation between ERα immunopositivity in the female hypothalamic VMN and female sexual receptivity, suggesting that expression of NHP female sexual receptivity is in direct proportion to ERα expression within a highly localized portion of the mediobasal hypothalamus.

In contrast to clear quantitative relationships between decrements in mediobasal hypothalamic ERα expression and diminished female marmoset sexual receptivity measures, such decrements in ERα expression do not lead to increased female sexual rejection of male partners. Certainly, deficits in E_2_ production from ovarian E_2_ depletion following OVX, as well as total E_2_ depletion by aromatase inhibitor [57], lead to loss of female sexual receptivity that is matched by increased female sexual rejection behavior toward the male partners. In those circumstances, however, increases in peripheral and/or hypothalamic androgen levels were shown to occur, while hyperandrogenism was absent in treatments employed in the present study. It may therefore be the case that circulating and/or locally produced androgens may serve as the primary trigger for the expression of sexual rejection behavior in female marmoset monkeys. It is also possible that lack of E_2_ signaling through the VMN (and possibly mediobasal hypothalamic) ERα contributes to inhibition of rejection behavior in a quantitative fashion, but only once a minimal threshold level is reached of either VMN ERα expression or E_2_ action via VMN ERα expression. Near complete ERα gene silencing in the rat VMN was found to produce significant increases in female sexual rejection behavior [32], presumably in the absence of altered androgen production. Our incomplete KD of ERα may thus be sufficient to disrupt sexual receptivity, but insufficient to disrupt the neural mechanisms mediating inhibition of sexual rejection. It is also possible, however, that E_2_ action via another VMN ER, or via ERα in another hypothalamic nucleus or neural location, inhibits female marmoset sexual rejection.

Delayed sexual responses in ERαKD female marmosets resembles female responses reported previously when examining ERα gene KD in the VMN of female rodents. In female rodents tested in traditional behavior testing arena environments (one male and one female rodent in a standard cage size), diminished ERα expression in the VMN resulted in a lack of both proceptive and receptive sexual behavior [32]. In more socially relevant, open, semi-natural behavioral testing environments, ERα gene KD in the VMN also reduces both proceptive and receptive sexual behaviors in female rodents, although a low frequency of sexually receptive behaviors are still observed [77]. The marmoset pairs used in the present study, however, were well-established male-female pairmates, and thus, the social dynamic was species appropriate. The marmoset testing arena is also not restricting of movement during behavioral testing sessions. Thus, the behavioral paradigm in the present study can be paralleled with the semi-natural rodent study [77], with similar results with respect to sexual behaviors.

In summary, these data suggest that, similar to female rodents, ERα expression in the VMN (or possibly MBH) of the female marmoset is the neurolocation-specific receptor mechanism regulating female sexual receptivity and responsiveness toward male partners, and likely in other NHPs and women. For the first time in an NHP, this study supports a major role for ERα in the VMN (MBH) in mediating female sexual receptivity and responsiveness.

### Mediobasal Hypothalamic Estrogen Receptor α and Female Reproductive Neuroendocrine Regulation of Luteinizing Hormone

Recent studies of ARC kisspeptin neurons in mice have provided evidence that ERα in these cells mediate at least some of the negative feedback actions of ovarian E_2_ on GnRH release. We therefore investigated the effect of MBH ERα KD on gonadotropin secretion, predicting that reductions in ER expression would be accompanied by relative resistance to E_2_-negative feedback effects. Measurement of plasma CG levels in ERαKD and control subjects revealed several significant differences between groups, with ERαKD females exhibiting a greater change from baseline relative to baseline, consistent with our hypothesis. No significant differences between groups were found, however, in mean plasma CG levels. The latter, however, appeared to mirror a progressive increase in CG values for the first 3 study months observed in CG change from baseline and in ERαKD females, alone. The modest, and perhaps transient, effect of ERα diminishment on plasma CG levels may reflect an insufficient KD of receptor expression to produce a substantial disruption of E_2_-negative feedback actions through MBH neurons. This is particularly likely considering the relatively modest extent of ERα reduction that was induced in the ARC (diminished intensity of cell ERα expression in contrast to diminished numbers of cells expressing ERα in the VMN), which is believed to be the primary locus of feedback mediated by resident kisspeptin neurons [78]. It is also possible that the frequency of GnRH pulsatility was altered in ERαKD animals, but any such changes could not be detected without the analysis of GnRH pulsatility. Such a result was recently obtained in ovary-intact female mice, in which sufficient KD (70%-80%) of ERα in ARC neurons by genomic editing, even on one side of the MBH alone, accelerated GnRH pulse generation that manifested in the circulation as LH pulses with low amplitude and erratic frequency that left mean LH levels unchanged or diminished [43]. The minor impairment of negative feedback regulation may also reflect a predominance of direct inhibitory actions of E_2_ via ERα expressed in pituitary gonadotropes. Classic studies in rhesus macaques demonstrated robust negative feedback effects of E_2_ at the level of the pituitary gland [79], and similar inhibitory effects of E_2_ appear to operate in marmosets [80]. As reported in female rodent studies when experimentally diminished or absent ERα expression is limited to within the hypothalamus, ERα expression is still maintained on gonadotropes in the anterior pituitary thus diminishing gonadotropin responsiveness [43].

### Mediobasal Hypothalamic Estrogen Receptor α and Regulation of Female Metabolic Function

Rodent studies have revealed that hypothalamic regulation of energy homeostasis is mediated by a neural network that includes neurons of the ARC, VMN, dorsomedial nucleus, paraventricular nucleus, and the lateral hypothalamic area [81]. The ARC and the VMN function as the primary sites for integrating hormonal cues such as leptin, insulin, ghrelin, and E_2_. While much is known of the specialized cells, receptors, and signaling pathways that mediate metabolic actions of E_2_ in rodents, the hypothalamic neuronal receptor mechanisms governing E_2_ regulation of female NHP metabolic function remain to be clarified. The actions of E_2_ via ERα on adiposity may occur directly in white adipose tissue, liver, muscle, and/or the pancreas [57, 82], as well as in hypothalamic neurons expressing ERα [28, 48]. The latter exert descending control over systemic organ systems via autonomic innervation [83, 84], including E_2_-induced alterations in food intake and energy expenditure, producing secondary metabolic states, or by a combination of these. Musatov and colleagues [45] demonstrated that viral vector–mediated ERα gene silencing in the VMN both of female mice and rats largely recapitulates a metabolic phenotype observed in whole-body ERαKO mice, including obesity, hyperphagia, impaired glucose tolerance, and reduced energy expenditure [45-52]. Stimulatory effects of E_2_ on energy expenditure are transduced in ERα-expressing neurons of the VMN of the hypothalamus [45], through mechanisms that include nonclassic ERα signaling [28] coupled to activation of PI3-kinase [46]. Hypometabolism and abdominal obesity, but not hyperphagia, are recapitulated in female mice lacking ERα in hypothalamic steroidogenic factor-1 (SF1) neurons of the VMN [48]. In contrast, deletion of ERα in hypothalamic POMC neurons in the ARC leads to hyperphagia, without directly influencing energy expenditure or fat distribution [85]. In the present study, we placed experimental and control monkeys on DIO to determine if diminished ERα expression in the VMN and/or ARC would result in an exacerbation of the metabolic effects of the obesogenic diet. We observed no statistically significant differences between the ERαKD and control groups over the 9-month treatment period in weight gain, fat accumulation, and locomotor activity, despite diminished calorie consumption and mild hyperglycemia in the ERαKD female group. DIO alone, however, contributed comparable and substantial gains both in female body weight (∼25%) and total body fat (∼50%) in both female groups. DIO may thus have overwhelmed contributions made by diminished E_2_ activity in the MBH. We also observed correlations between ERα immunopositivity and calorie consumption in the ventral VMN (positive) and the ventral ARC (negative), suggesting that expression of female NHP food intake is in direct proportion to ERα expression in analogous hypothalamic locations as that demonstrated in female rodents, the ventrolateral VMN [45, 48] and the ARC [54, 86-88].

In view of the incompleteness of ERα gene silencing achieved in this study, we can only conclude on the basis of these results that moderate reduction in ERα signaling in the MBH does not substantially affect metabolic parameters in female monkeys placed on DIO. As with our observations with respect to feedback regulation of gonadotropin secretions, it remains unclear whether different signaling mechanisms mediate E_2_ metabolic actions in the marmoset vs mouse hypothalamus, or if our experimental procedures were insufficient to reveal any similarities in metabolic functions of hypothalamic ERα in this NHP. Some diminution in energy expenditure occurred in ERαKD monkeys, nevertheless, for them to match the weight gain of controls on fewer calories consumed despite being allotted comparable increments in chow provided daily.

Diminished ERα expression in the VMN and ARC was found to be accompanied by a slight, but statistically significant, increase in serum glucose levels during a GTT. Complete ERα deletion as well as ERα gene silencing in the VMN in female mice produce a metabolic syndrome that includes hyperglycemia, glucose intolerance, and insulin insensitivity [45-52]. Deletion of ERα in SF1 neurons likewise leads to impaired glucose control, as does the depletion of mGluR5 from SF1 neurons [89]. These effects may be mediated in part by modulation of neural signals conveyed via descending autonomic pathways, ultimately altering hepatic vagal tone and hence, hepatic insulin sensitivity. It remains to be confirmed whether this circuit operates similarly in NHPs to mediate the glucoregulatory effects of E_2_ through ERα.

### Limitations

Our gene-silencing approaches emulate previous studies in female rats examining the role of ERα in discrete nuclei of the hypothalamus [28-36, 45-52]. Compared with rodent approaches, however, we employed within-surgery MRI guidance to refine targeting of the VMN and ARC in each individual female, as NHPs have notable individual variations in neuroanatomical locations [73, 76] in contrast to laboratory rats and mice. This between-NHP anatomical variation may therefore have contributed to our between-female variation in ERαKD KD achieved. We also cannot assess the relative loss of ERα within a discrete hypothalamic nucleus within each female NHP in the ERαKD group as we had no ability to assess ERα expression in the hypothalamus at baseline. Moreover, our immunohistological assessments of ERα expression were performed approximately 10 to 12 months after KD procedures, 4 to 6 months after sexual behavior testing sessions as well as the GTT, and 2 to 4 months after assessment of calorie consumption. Thus, we do not know if our gene-silencing procedures produced a stable level of ERα diminishment that was maintained over the course of the experiments, or if waning of the KD effectiveness occurred over time, leading us to underestimate the extent to which ERα was reduced at the time of our behavioral, metabolic, and endocrine phenotyping. In addition, while we cannot exclude neuronal dysfunction caused by an off-target effect of shRNA, this scenario is unlikely given that the selected ER target sequence does not match any other marmoset monkey messenger RNA in the GenBank database. There were also no baseline data obtained for several parameters, including sexual behavior, calorie consumption, body composition, locomotory activity, and glucoregulation, so we cannot eliminate between-group differences prior to onset of this study.

### Summary

The present findings confirm that sufficient ERα activation in MBH neurons is obligatory for species-typical expression of sexual receptivity in female marmoset monkeys. Thus, this critical role for hypothalamic ERα is clearly conserved among rodents and at least one primate species. Some support for ERα involvement in macaque sexual behavior was previously provided by the finding that mating activates ERα-expressing neurons in the VMN, as reported by c-Fos expression in the VMN and POA of female cynomolgus macaques [90]. To our knowledge, however, no behavioral studies have been conducted to assess the behavioral effects of ERα or ERβ-specific agonists on sexual behavior in any female NHP. Likewise, it remains to be determined if ERα in VMN neurons play a similarly critical role in the support of sexual motivation in women. In 2013, Quaynor and colleagues [91] identified complete estrogen insensitivity in a female patient with a homozygous loss-of-function *ESR1* variant. While noted to have delayed puberty, absent breast development, and elevated serum estrogen and LH levels, as well as polycystic ovaries, no psychological findings were reported [91]. Similar hormonal and growth phenotypes were reported in 2 additional female patients with inactivating *ESR1* mutations [92]. Nevertheless, ERα-regulated dopaminergic, adrenergic, and serotoninergic neuronal circuitries control female sexual behavior in nonprimates and NHPs [92-94]. A recently Food and Drug Administration–approved pharmaceutical, flibanserin, a postsynaptic serotonin 1A receptor agonist and postsynaptic serotonin 2A receptor antagonist [92, 93], with prefrontal cortex action causing downstream release of dopamine and norepinephrine and reduction of serotonin [93], improves appetitive sexual behavior in female rodents [95], sexual interest and desire in women [96, 97], and social intimacy in female NHPs [94]. Further exploration of the roles played by neural ERα in supporting female sexual behavior in NHPs and women may provide more efficacious clinical management of hypoactive sexual desire disorder.

## Data Availability

Some or all data sets generated during and/or analyzed during the present study are not publicly available but are available from the corresponding author on reasonable request.

## Abbreviations

3D: 3-dimensional
17-OHP4: 17-hydroxyprogesterone
AAV: adeno-associated virus
ARC: arcuate nucleus
ARCd: arcuate nucleus dorsal
ARCv: arcuate nucleus ventral
AUC: area under the curve
BMC: bone mineral content
BMD: bone mineral density
CG: chorionic gonadotropin
CMV: cytomegalovirus
DHEA: dehydroepiandrosterone
DIO: diet-induced obesity
DXA: dual-energy x-ray absorptiometry
E2: estradiol
EIA: enzyme immunoassay
ERα: estrogen receptor α
ERαKD: estrogen receptor α knockdown
FFM: fat free mass
GFP: green fluorescent protein
GnRH: gonadotropin-releasing hormone
IM: intramuscular
ir: immunoreactive
KD: knockdown
KO: knockout
LC-MS/MS: liquid chromatography–tandem mass spectrometry
LET: letrozole
LH: luteinizing hormone
MBH: mediobasal hypothalamus
mPOA: medial preoptic area
MRI: magnetic resonance imaging
NHP: nonhuman primate
OGTT: oral glucose tolerance test
OVX: ovariectomy
POA: preoptic area
POMC: pro-opiomelanocortin
RC: rostral-caudal
RNAi: RNA interference
ROI: region of interest
SC: subcutaneous
SF1: steroidogenic factor-1
shRNA: short hairpin RNA
siRNA: small interfering RNA
VMN: ventromedial nucleus
VMNm: ventromedial nucleus mid
VMNv: ventromedial nucleus ventral
